# CD39 and CD73: biological functions, diseases and therapy

**DOI:** 10.1186/s43556-025-00345-9

**Published:** 2025-11-05

**Authors:** Jie Shen, Bin Liao, Li Gong, Sha Li, Juan Zhao, Huiyao Yang, Yi Gong, Yongsheng Li

**Affiliations:** Phase I Clinical Trial Ward, Chongqing University Cancer Hospital, 181 Hanyu Road, Shapingba District, Chongqing, 400030 China

**Keywords:** CD39, CD73, Adenosine, Functions, Diseases, Therapy

## Abstract

Cluster of differentiation 39 (CD39) and CD73 are ectonucleotidases that play pivotal roles in purinergic signaling. CD39 catalyzes the hydrolysis of adenosine triphosphate (ATP) to adenosine diphosphate (ADP) and subsequently to adenosine monophosphate (AMP), while CD73 further catalyzes the hydrolysis of AMP to adenosine. These ectonucleotidases are expressed across diverse cell types and exhibit pleiotropic functions in immune regulation, physiological homeostasis, and disease pathogenesis. Recent preclinical studies have increasingly identified CD39 and CD73 as promising therapeutic targets in various disease states, particularly in cancer. This review provides a comprehensive summary of the current advancements in CD39 and CD73 research, emphasizing their structural characteristics, distribution, enzymatic and non-enzymatic activities, as well as their biological functions. We discuss the involvement of CD39 and CD73 in multiple disease states, including cancer, autoimmune disorders, inflammatory diseases, cardiovascular disorders, infectious diseases, and neurological disorders. Furthermore, we present existing preclinical and clinical research on reported CD39 and CD73 inhibitors, which include small-molecule inhibitors, antibodies, advanced delivery systems, and combinations with adenosine receptor antagonists, targeted therapy, immunotherapy, and chemotherapy, thereby providing a foundation for future investigations. The anti-tumor efficacy of these inhibitors, observed across various tumor types, is primarily mediated through adenosine-dependent mechanisms. Despite these encouraging preclinical findings, several challenges hinder the application of CD39 and CD73 inhibitors. It is essential to optimize and modify their structures, enhance dosage forms, and adjust both the dosage and timing of administration to achieve high selectivity while minimizing off-target effects. Future research is anticipated to concentrate on mechanistic exploration and rational drug design, while also broadening their therapeutic potential to encompass additional diseases.

## Introduction

Accumulating evidence highlights the significant roles of the purinergic pathway in both physiological homeostasis and pathological processes, with ectonucleotidases serving as key components within this pathway [1]. Ectonucleotidases are enzymes found on the cell surface that catalyze the hydrolysis of extracellular nucleotides, including adenosine triphosphate (ATP), adenosine diphosphate (ADP), adenosine monophosphate (AMP), uridine triphosphate (UTP), uridine diphosphate (UDP), and nicotinamide adenine dinucleotide (NAD) [2]. These enzymes are classified into several categories, including nucleoside triphosphate diphosphohydrolases (NTPDases), nucleotide pyrophosphatase/phosphodiesterases (NPPs), ecto-5'-nucleotidase, and alkaline phosphatases [3]. The NTPDase family is further divided into eight members, NTPDase1-8, based on their distinct subcellular localization patterns and substrate specificities [4]. Seven human 5′-nucleotidase enzymes have been identified: one is located in the mitochondrial matrix, one is located on the outer surface of the cell membrane, while the others are cytosolic enzymes [5]. These 5′-nucleotidases exhibit variations in substrate affinities, as well as differences in the size and arrangement of their quaternary structures.

The ectonucleotidases cluster of differentiation 39 (CD39) and CD73 play a crucial role in modulating purinergic signaling by regulating the levels of pro-inflammatory ATP and immunosuppressive adenosine [6]. CD39, also known as ectonucleoside triphosphate diphosphohydrolase 1 (ENTPDase1 or ENTPD1), is a cell surface glycoprotein that belongs to the NTPDase family and catalyzes the sequential hydrolysis of ATP to ADP and then to AMP [7]. CD73, also referred to as ecto-5′-nucleotidase (e5NT or NT5E), is a glycosylphosphatidylinositol (GPI)-anchored glycoprotein located on the cell surface that mediates the conversion of AMP into extracellular adenosine (eADO) [8]. Furthermore, eADO interacts with four G-protein-coupled adenosine receptors: A1R, A2AR, A2BR, and A3R [9]. A1R and A3R are coupled to Gi and Go proteins, which suppress adenylate cyclase activity and decrease cyclic AMP (cAMP) levels. In contrast, A2AR and A2BR are coupled to Gs proteins, stimulating adenylate cyclase and increasing cAMP levels. cAMP plays a crucial role in the protein kinase A (PKA)-mediated phosphorylation of cAMP-response element binding protein [10]. Additionally, adenosine receptors engage with the phosphatidylinositol 3-kinase/protein kinase B (PI3K/AKT) and mitogen-activated protein kinase (MAPK) signaling pathways. The expression of adenosine receptors varies across different cell types. Notably, the expression of adenosine receptors on macrophages and dendritic cells (DCs) undergoes dynamic changes during their development and activation [11]. On T cells, A2AR and A2BR are primarily responsible for the immunosuppressive effects of eADO, thereby facilitating immune escape [12, 13].

CD39 and CD73 serve as pivotal regulators of cellular homeostasis, orchestrating stress responses, modulating injury processes, and participating in disease mechanisms. Under homeostatic conditions, both CD39 and CD73 are expressed on endothelial cells. Additionally, CD39 is found on platelets, while CD73 is present on mesenchymal and stromal cells [14–16]. Additionally, CD39 and CD73 are expressed on various immune cells, including T cells, B cells, natural killer (NK) cells, DCs, and macrophages [17–22]. Their expressions are regulated by multiple microenvironmental factors, such as pro-inflammatory cytokines, oxidative stress, and hypoxic conditions [23]. Notably, these proteins are also expressed on a wide range of tumor cells, including those from lung cancer, gastric cancer, ovarian cancer, and breast cancer [24–28].

Emerging evidence suggests that CD39 and CD73 play multifaceted roles in inflammatory disorders, cardiovascular diseases, and infectious diseases, particularly in cancer [29–33]. The elevated concentrations of eADO within the tumor microenvironment (TME) primarily arise from ATP hydrolysis, which is catalyzed by the sequential actions of CD39 and CD73 [34]. The inhibition of CD39 and CD73 promotes anti-tumor immunity by modulating the processes such as tumor cell proliferation, migration, and angiogenesis. These effects are mediated through increased accumulation of extracellular ATP and decreased production of eADO [35–38]. Consequently, CD39 and CD73 have emerged as promising therapeutic targets in cancer treatment, spurring the discovery and development of an increasing number of inhibitors. These initiatives have significantly advanced our understanding of their biological functions and therapeutic potential.

In this review, we provide a comprehensive overview of recent advances in CD39 and CD73 research, emphasizing their pivotal roles in purinergic signaling pathways. We present recent discoveries regarding the biology of CD39 and CD73, which encompass their structure, distribution, enzymatic properties, non-enzymatic activities, and biological functions. Furthermore, we discuss the current understanding of CD39 and CD73 in disease pathogenesis, particularly their involvement in cancer, autoimmune diseases, inflammatory diseases, cardiovascular diseases, infectious diseases, and neurological disorders. Additionally, we offer a thorough overview of therapeutic strategies targeting CD39 and CD73, including small molecule inhibitors, antibodies, and advanced drug delivery systems. Beyond monotherapy approaches, we analyze combination strategies that integrate CD39 and CD73 inhibitors with other treatment modalities, thereby providing insights for future research directions.

## Nucleotide and adenosine metabolism

**Fig. 1:**
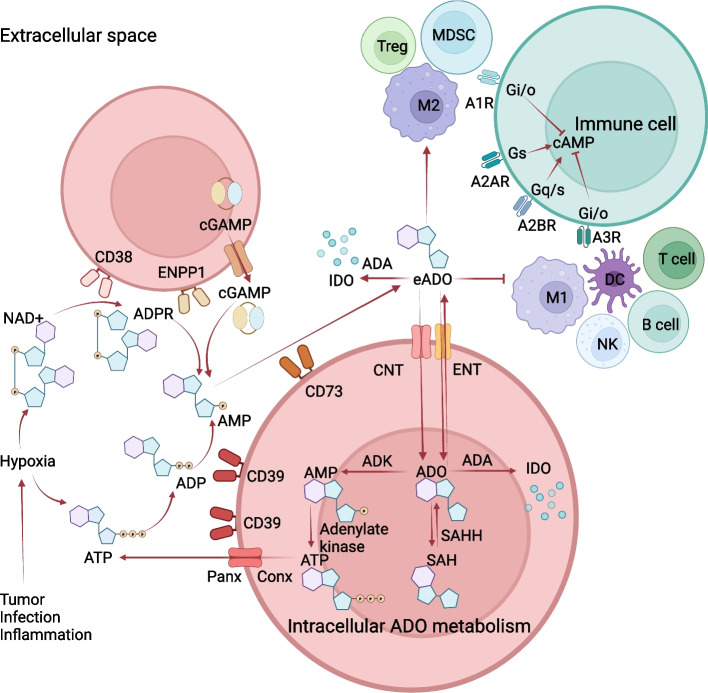
CD39 and CD73 in the ATP-ADO pathway. Hypoxic conditions, commonly found in TMEs, as well as in infection or inflammatory sites, stimulate the extracellular release of ATP, NAD^+^ and cGAMP. Subsequently, eADO is generated through the enzymatic activities of CD39, CD73, CD38, and ENPP1. The transport of eADO is facilitated by concentrative nucleoside transporters (CNT) and equilibrative nucleoside transporters (ENT). ADO is converted back into ATP via adenosine kinase (ADK) and adenylate kinase, after which ATP is released into the extracellular space through connexin (Conx) and pannexin (Panx) channels. Additionally, ADO is transformed into S-adenosylhomocysteine (SAH) by S-adenosylhomocysteine hydrolase (SAHH). Both ADO and eADO are further catabolized to inosine (INO) by adenosine deaminase (ADA). Furthermore, eADO exerts an inhibitory effect on the activity of NK cells, T cells, B cells, classical (M1) macrophages, and dendritic cells (DCs), while simultaneously enhancing the activity of MDSCs, alternative (M2) macrophages, and Tregs through interactions with A1R, A2AR, A2BR, and A3R receptors. This interaction results in elevated levels of cAMP, ultimately promoting an immunosuppressive environment

CD39 and CD73 play crucial roles in mediating immunosuppression through the adenosine pathway. As illustrated in Fig. 1, ATP is released into the extracellular environment during hypoxia. Initially, ATP is degraded to ADP by CD39, which is then hydrolyzed to AMP. Subsequently, AMP is further hydrolyzed into eADO by CD73 [17]. In addition to ATP, other nucleotides can be converted to eADO through a CD39-independent pathway that involves the ectonucleotidases CD38 and ectonucleotide pyrophosphatase/phosphodiesterase 1 (ENPP1, also known as CD203a or PC-1). This pathway enables cancer cells to evade immune surveillance and promotes metastasis [39]. CD38, a predominant NADase, facilitates the metabolism of NAD^+^ into cyclic ADP-ribose (cADPR) and ADP-ribose (ADPR), and it hydrolyzes cADPR into ADPR [40]. ENPP1 generates AMP by hydrolyzing various substrates: it can hydrolyze ATP or ADPR to yield AMP and inorganic pyrophosphate (PPi), and it can also hydrolyze NAD ^+^ to produce AMP and nicotinamide mononucleotide (NMN) [39]. The generated AMP is subsequently converted to eADO by CD73. In the acidic and hypoxic environment of the bone marrow niche, the CD38-ENPP1-CD73-mediated eADO pathway is more effective than the canonical pathway, which accounts for the observed antitumor effects of CD38 inhibition in myeloma [41]. Furthermore, the metabolism of extracellular cyclic GMP-AMP (cGAMP) represents another pathway for adenosine generation in cancer [42]. cGAMP is synthesized by cyclic GMP-AMP synthase (cGAS) upon binding to cytosolic DNA. It subsequently interacts with the stimulator of interferon genes (STING), leading to the transcription of pro-inflammatory cytokines through the activation of IRF3 and NF-κB [43]. After being exported to the extracellular space via gap junctions and transporters, cGAMP is hydrolyzed into AMP and GMP by ENPP1, while AMP is converted into adenosine by CD73. This process transforms an immunostimulatory pathway into an immunosuppressive one, thereby contributing to tumor progression.

The extensive network of enzymes—including ATP-regenerating nucleoside diphosphate kinases (NDPK), adenylate kinase (AK), and nucleoside-inactivating enzymes such as adenosine deaminase (ADA) and purine nucleoside phosphorylase (PNP)—works in concert with CD39, CD73, and ENPP1 to coordinate the control of extracellular nucleotide and nucleoside levels [43–45]. NDPK facilitates the interconversion of ATP and ADP, as well as NTP and NDP, while AK1 catalyzes the reversible transformation between ADP and ATP and between ADP and AMP, respectively [44]. ADA and PNP are involved in the metabolism of eADO: ADA converts eADO to inosine (INO), and PNP subsequently converts INO to hypoxanthine (HYP), which is ultimately metabolized to uric acid *via* xanthine. Additionally, eADO is transported into the intracellular space by concentrative nucleoside transporters (CNT) and equilibrative nucleoside transporters (ENT). The metabolism of intracellular adenosine is regulated by phosphorylating enzymes, including adenosine kinase (ADK), AK1, and NDPK, as well as inactivating enzymes such as ADA and PNP [45]. In the phosphorylation pathway, intracellular adenosine is converted to AMP by ADK, followed by the conversion of AMP to ADP by AK1, and subsequently, ADP is converted to ATP by NDPK. ATP is released into the extracellular space through connexin (Conx) and pannexin (Panx) channels [46]. In the inactivation pathway, intracellular adenosine is converted to INO by ADA, which is then transformed into HYP by PNP. Furthermore, S-adenosylhomocysteine hydrolase (SAHH) facilitates the interconversion between intracellular adenosine and S-adenosylhomocysteine (SAH), with SAH being hydrolyzed into adenosine and L-homocysteine (HCy).

Furthermore, CD73 plays a crucial role in NAD metabolism. Initially, extracellular NMN is degraded into nicotinamide riboside (NR) by CD73. NR then enters the cytoplasm, where it is phosphorylated into NMN through the action of nicotinamide riboside kinase 1 (NRK1). Subsequently, NMN is converted into NAD. Additionally, CD73 enhances the intracellular ratio of NAD to NADH by promoting glycolysis, which in turn increases the activity of malate dehydrogenase (MDH). This process facilitates the degradation of oxaloacetate by MDH, resulting in the synthesis of aspartate and subsequently promoting tumor growth and proliferation [47].

Importantly, eADO demonstrates significant anti-inflammatory and immunosuppressive effects through its interaction with adenosine receptors [17]. eADO can inhibit the proliferation of NK cells and T effector cells, thereby fostering a pro-tumor environment [10]. The TME is characterized by hypoxia and low pH levels, with elevated concentrations of eADO persisting over time. Upon activation of A2AR and other adenosine receptors, eADO is transported into cells and metabolized, thereby contributing to the attenuation of immune responses and the prevention of tumor invasion *via* receptor-independent pathways [45]. Preclinical studies indicate that eADO inhibits anti-tumor T cell activity by binding to A2AR, while simultaneously enhancing the immunosuppressive functions of tumor-associated fibroblasts and myeloid cells through its interaction with A2BR [48, 49]. Recent findings suggest that the anti-tumor effects of adenosine are mediated by the modulation of pyrimidine metabolism in cytotoxic T cells [50, 51]. As an adenosine transporter, ENT1 facilitates the uptake of adenosine, thereby promoting immunosuppression and pyrimidine starvation [50]. By restoring pyrimidine nucleotide synthesis in T cells that are suppressed by adenosine, ENT1 inhibition subsequently enhances anti-tumor T cell responses [51].

## Biology of CD39 and CD73

CD39 and CD73 play crucial roles in regulating extracellular ATP hydrolysis and eADO production. Their expression patterns across immune cells, fibroblasts, and tumor cells, along with the existence of both membrane-bound and soluble forms, highlight their multifaceted regulatory potential. Beyond their well-established immunoregulatory functions, these ectonucleotidases exhibit pleiotropic effects in vascular homeostasis, neuroregulation, bone metabolism, adipose tissue metabolism, and stress response regulation, thereby facilitating their involvement in complex intercellular communication networks in both physiological and pathological contexts.

### Structure and distribution

The cell surface glycoprotein CD39, comprising a sequence of 510 amino acids (70–100 kDa), contains 11 conserved cysteine residues and features two transmembrane domains situated near both the N-terminal and C-terminal ends [52]. Additionally, it possesses seven N-glycosylation sites, which are crucial for maintaining its structural integrity and functional activity. The extracellular domain is characterized by five conserved ATPase-conserved regions (ACRs) [53]. Notably, the cysteine at position 13 within the N-terminal intracytoplasmic domain of CD39 is palmitoylated, a modification that may influence the protein's attachment to the membrane. Supporting this hypothesis, truncation of the N-terminal leads to the production of soluble CD39 [7]. The crystal structure of a soluble variant of CD39 reveals that the asymmetric unit consists of four polypeptide chains (labeled A to D). In chains B and D, the N374 glycosylation loop is well-defined, and three chloride ions occupy the active-site clefts, one of which binds to the phosphate-binding loop of ACR4. This observation underscores the capacity of ACR4 loops to attract negatively charged molecules [54].

CD73 is a homodimeric enzyme composed of 548 amino acids (~ 70 kDa) [8, 55]. The crystal structure reveals that CD73 exists as a non-covalent dimer, with each subunit composed of an N-terminal and a C-terminal domain interconnected by a hinge region [56]. The N-terminal domain contains binding sites for zinc and cobalt ions and is responsible for phosphohydrolase activity, while the C-terminal domain includes the substrate binding site and is anchored to the cell membrane *via* a GPI anchor [56, 57]. Additionally, the hinge region facilitates domain movement, leading to distinct conformations: open I, open II, closed III, and closed IV, with the active site located at the interface between the two domains in the closed conformation [58]. The substrate binding domain of CD73 consists of hydrophobic residues (Phe-417, Phe-500) and charged residues (Arg-354, Arg-395, Asn-390) [59]. Soluble CD73 exists in a homodimeric configuration, with C-terminal hydrogen bonds and hydrophobic interactions serving as crucial determinants of its stability. Notable characteristics include a disulfide bridge, three short amino acid stretches, various oligosaccharide modifications, and an active site where the purine ring of the substrate is positioned between Phe417 and Phe500 [57].

CD39 and CD73 are expressed on various types of vascular endothelial cells, including brain and vein endothelial cells [8, 60–62]. In CD73-deficient human umbilical vein endothelial cells, there is an upregulation of surface expression of leukocyte adhesion molecules, an increase in stress fiber formation, and enhanced endothelial permeability [62]. Given that the endothelium is a crucial component of the blood–brain barrier, its permeability is known to increase during hypoxia and ischemia. Consequently, the adenosine produced by CD39 and CD73 on vascular endothelial cells plays a vital role in protecting tissues from such insults [63]. Although CD73 is expressed by both vascular and lymphatic endothelial cells, it specifically promotes endothelial barrier capacity and sprouting only within the blood vasculature [64]. Furthermore, the adenosine generated by CD73 in vascular endothelial cells serves as a paracrine signal that orchestrates de novo lipogenesis in white adipose tissue [14].

CD39 and CD73 are expressed across a diverse array of immune cell populations, including T cells, B cells, NK cells, DCs, and myeloid-derived suppressor cells (MDSCs) [17, 19, 20, 23, 65, 66]. Additionally, monocytes and neutrophils have been reported to express CD39 [67]. The expression of CD39 on CD8^+^ T cells is linked to T cell exhaustion in cancer [68]. CD39 expression on CD4^+^ T cells may serve as a reliable marker for bystander CD4^+^ T cells [69]. A small subset of γδ T cells co-expresses both CD39 and CD73 [70]. CD39 and CD73 expression is also observed on regulatory T cells (Tregs) and memory T cells [17, 71, 72]. While CD73 is present on naive and memory CD8^+^ T cells, its expression diminishes in terminal effector differentiation [73]. Notably, migratory Tregs exhibit significantly higher levels of CD39 and CD73 expression compared to their non-migrating counterparts [74].

CD39 and CD73 have been identified as being overexpressed in various tumor cells, including those from gastric cancer, ovarian cancer, cholangiocarcinoma, lung cancer, and bladder cancer [24–27, 75, 76]. Furthermore, non-small cell lung cancer (NSCLC) characterized by mutations in the epidermal growth factor receptor (EGFR) and Kirsten rat sarcoma viral oncogene homolog (KRAS), displays pronounced CD73 expression signatures [77]. Additionally, CD73 is prominently expressed in DCs, stromal cells, lymphocytes, and mesenchymal stem cells within the context of multiple myeloma [78]. In gastric cancer, CD39 is present on tumor-infiltrating lymphocytes (TILs) [25]. In ovarian cancer, CD39 is expressed on tumor-associated macrophages (TAMs), whereas CD73 is expressed on stromal fibroblasts [79]. In glioblastoma, microglia predominantly express CD39, whereas tumor cells express CD73 [80].

CD39 and CD73 are expressed in various fibroblast populations [49, 81, 82]. Specifically, CD39 is identified in cardiac fibroblasts, while CD73 is predominantly expressed in cancer-associated fibroblasts (CAFs). Both ectonucleotidases are co-expressed in gingival fibroblasts. Moreover, CD73 serves as the principal ocular ectonucleotidase, exhibiting selective expression in the photoreceptor layer. In contrast, CD39 is prominently expressed in the optic nerve head, microglia, neuronal processes, retinal vasculature, and corneal tissues [83].

### Enzymatic and non-enzymatic activity

#### Enzymatic activity and substrate specificity

CD39 hydrolyzes extracellular ATP and ADP to AMP but is unable to further degrade AMP. Additionally, CD39 facilitates the conversion of UTP and UDP into UMP, with UDP being metabolized only after significant depletion of UTP levels [4]. Comparative kinetic analysis indicates that CD39 follows Michaelis–Menten kinetics and exhibits a greater catalytic preference for ATP/ADP compared to UTP/UDP. Linear reaction kinetics are observed for at least the first 30 min when using either ATP or ADP as substrates. Molecular dynamics simulations demonstrate that soluble CD39 displays substrate inhibition when ADP or ATP is utilized as substrate, with reaction rates initially peaking before declining at higher concentrations. In contrast, mild inhibition is observed with UTP and GTP at elevated concentrations, whereas no substrate inhibition is evident with UDP, GDP, or 2-MeS-ADP. These results indicate that substrate inhibition is substrate-specific and depends on both the nucleotide base and the number of phosphates [84]. Furthermore, divalent cations Mg^2^⁺ and Ca^2^⁺ are essential for CD39-mediated nucleotide hydrolysis. Although CD39 shows no distinct preference between these divalent cations, their concentrations significantly influence enzymatic activity, with optimal performance observed at concentrations of 1–5 mM. CD39 maintains sustained high activity within a narrower pH range (pH 7 ~ 9.5) under neutral to alkaline conditions, as indicated by activity thresholds exceeding 50% of maximal activity [4]. A comparative analysis of extracellular ATPases within the TCGA database reveals that CD39 is the predominant enzymatic player in the TME. It consistently demonstrates higher expression levels and superior enzymatic activity compared to ENPP1 and TNAP under slightly acidic conditions (pH 6.8) characteristic of the TME [38].

CD73 exhibits a substrate preference for AMP hydrolysis among the 5’-nucleoside monophosphates (AMP, CMP, UMP, IMP, and GMP), demonstrating characteristic Michaelis–Menten kinetics for AMP [85]. An evaluation of substrate specificity reveals the following activity profile: AMP > UMP > CMP > dAMP > dTMP > dCMP > IMP > GMP > dGMP. CD73 shows distinct base preferences and exhibits significantly lower activity with deoxyribonucleotides compared to their ribonucleotide monophosphate equivalents. Notably, several 2-substituted AMP derivatives, including 2-cyclopentylthio-AMP, 2-hexylthio-AMP, 2-cyclohexylethylthio-AMP, and 2-cyclohexylmethylthio-AMP, have been identified as substrates for CD73, which enzymatically converts them into their respective 2-substituted adenosine derivatives [86]. While AMP remains the preferred substrate, CD73 also demonstrates enzymatic activity toward NMN and NAD, albeit with significantly lower catalytic efficiency [87]. Furthermore, CD73-mediated AMP hydrolysis requires divalent cations, particularly Mg^2+^, and exhibits optimal activity at pH 9.5 within the range of pH 7 ~ 10.5, indicating a preference for alkaline conditions under these experimental circumstances [88].

#### Non-enzymatic activity

Beyond their established enzymatic activities, CD73 also exhibits non-enzymatic functions within cells. These non-enzymatic roles of CD73 play a crucial part in cellular adhesion and migration. In U138MG glioma cells, CD73 enhances cell adhesion through its interaction with the extracellular matrix [89]. Additionally, the overexpression of CD73 enhances cell proliferation and migration in cervical cancer, independent of its enzymatic activity, as the inhibition of enzymatic function does not reverse these effects [90]. Furthermore, the non-enzymatic functions of CD73 are significantly involved in endothelial tube formation, as demonstrated by the fact that pharmacological inhibition of its enzymatic activity does not disrupt this process [91]. In contrast, the non-enzymatic activities of CD39 remain largely unexplored.

### Biological functions

**Fig. 2:**
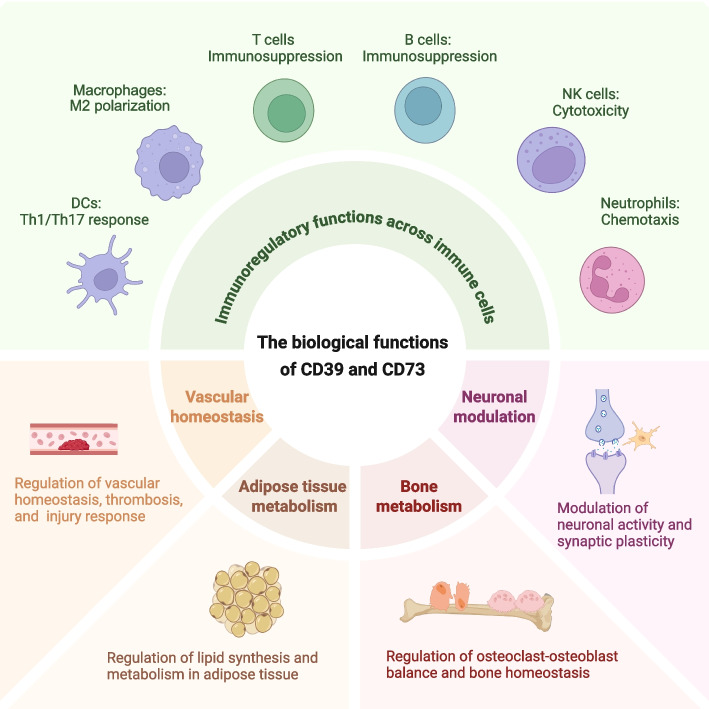
The biological functions of CD39 and CD73. CD39 and CD73 play a pivotal role in regulating immune responses by enhancing the immunosuppressive functions of T cells and B cells, boosting the cytotoxicity of NK cells, promoting the polarization of M2 macrophages, modulating DC-mediated Th1/Th17 responses, and regulating neutrophil chemotaxis. In addition to their immunoregulatory functions, CD39 and CD73 are also essential for neuronal modulation, vascular homeostasis, bone metabolism, and adipose tissue metabolism

CD39 and CD73 exert diverse biological functions through their enzymatic activities. As illustrated in Fig. 2, they play pivotal regulatory roles across various immune cell populations, including T cells, B cells, NK cells, neutrophils, macrophages, and DCs. In addition to their immune functions, CD39 and CD73 are also essential for neuronal modulation, vascular homeostasis, bone metabolism, and adipose tissue metabolism.

#### Immunoregulatory functions

The expression of CD39 and CD73 on Tregs, along with the expression of A2A receptors on effector T cells, establishes an immunosuppressive feedback loop [17]. CD73 expression on Tregs is upregulated through hypoxia-inducible factor 1 alpha (HIF-1α)-mediated signaling [92]. In contrast, CD39 expression on Tregs is regulated by transforming growth factor-beta (TGF-β)/mechanistic target of rapamycin (mTOR) signaling and autophagy activation mediated by reactive oxygen species (ROS) [93]. Human CD39^high^ Tregs demonstrate enhanced stability under inflammatory conditions, while CD39^low^ Tregs tend to transdifferentiate into T helper cells (Th1/Th17) [94]. CD39^+^ conventional CD4^+^ T cells display signs of exhaustion and cytotoxic potential, as evidenced by elevated levels of interferon (IFN)-γ, granzyme B, perforin, and CD107a, while exhibiting reduced tumor necrosis factor (TNF) levels [95]. In addition, CD39^+^ CD8^+^ T cells show superior functionality, characterized by increased IFN-γ and CD107a levels [96].

B cells play a multifaceted role in immune responses, primarily through the production of antibodies, the presentation of antigens, and the secretion of cytokines. Interleukin (IL)−10 has been recognized as a significant mediator of B cell-induced immunosuppression, the discovery of CD73-mediated eADO production *via* an IL-10-independent mechanism highlights a novel regulatory function of B cells [18]. A regulatory subset of eADO-producing CD39^+^ CD73^+^ B cells demonstrates immunosuppressive properties. Upon in vitro activation, these CD39^high^ B cells proliferate through A1R/A2AR-mediated autocrine signaling, upregulate CD73 expression, and, when co-cultured with autologous effector T cells, suppress T cell activation and proliferation while secreting elevated levels of IL-6 and IL-10 [97].

NK cells are recognized for the ability to secrete a diverse range of cytokines, exert cytotoxic effects, and regulate immune responses. Circulating CD39^+^ NK cells exhibit significantly enhanced cytotoxic activity, as evidenced by their expression of CD107a, TNF-α, and IFN-γ following stimulation with IL-12, IL-18, or NK-sensitive K562 cells [19]. IL-15 is a potent inducer of CD39^+^ NK cell production, while the A2AR limits this process by inhibiting IL-15-induced pathways [19]. Typically, NK cells do not express CD73 in healthy tissues and blood. In the context of cancer, CD73 expression on NK cells is generally low, tumor-specific, and influenced by the surrounding microenvironment. Notably, CD73^+^ NK cells demonstrate enhanced functionality in vitro [98].

Neutrophils are essential effector cells in innate immunity, providing defense against bacterial and fungal infections. CD39 regulates neutrophil chemotaxis, associating with the leading edge of migrating neutrophils. Both genetic knockout and pharmacological inhibition of CD39 impair the chemotactic capacity of these cells [99]. Additionally, CD39 and CD73 play critical roles in regulating neutrophil transmigration, thereby preventing excessive pulmonary accumulation after lipopolysaccharide-induced lung injury [100]. Moreover, low levels of adenosine activate A1R and A3R, enhancing neutrophil chemotaxis and phagocytosis, while higher levels engage A2AR and A2BR, which suppress the release of inflammatory mediators, oxidative burst, and degranulation in neutrophils [101].

Macrophages can polarize into pro-inflammatory (M1) or anti-inflammatory (M2) phenotypes, which are crucial for the initiation, progression, and resolution of inflammation. M1 macrophages exhibit lower expression of CD39 and CD73 compared to M2 macrophages, resulting in ATP accumulation in the former, while M2 macrophages rapidly convert ATP to eADO [102]. CD39 is critical for regulating P2X7-dependent macrophage responses; macrophages lacking CD39 demonstrate a loss of ADPase activity and reduced ATPase activity [21]. Furthermore, CD73 plays a crucial role in modulating the pro-inflammatory responses of macrophages. The inhibition or down-regulation of CD73 during infection results in an increased release of pro-inflammatory cytokines and nitric oxide (NO), which in turn enhances bacterial clearance and improves host survival [103].

DCs collectively modulate the balance between effector T cells and Tregs. The expression of CD39 induced by IL-27 in DCs reduces ATP levels, which inhibits the differentiation of Th1 and Th17 cells, ultimately attenuating T-cell responses and autoimmune reactions [104]. CD73^+^ DCs are essential for Th17 responses, as they convert AMP to eADO [105]. Furthermore, the expression of CD73 on skin DCs plays a critical role in modulating contact hypersensitivity responses. The eADO produced by CD73 inhibits the migration of Langerin^+^ DCs from the skin to the draining lymph nodes after sensitization [106].

#### Systemic homeostatic functions

In addition to their immunoregulatory roles, CD39 and CD73 play vital roles in maintaining vascular homeostasis, regulating thrombosis, and mediating responses to vascular injury. Notably, these molecules are expressed in rat vascular smooth muscle cells, where their production of eADO counteracts ATP-mediated vasoconstriction [107]. In CD39-deficient mice, the administration of UDP/UTP leads to sustained aortic constriction, highlighting the importance of CD39 for maintaining vascular tone [108]. CD39, which is present on both platelets and endothelial cells, inhibits thrombosis by synergizing with prostacyclin, NO, and heparan sulfate [15]. Importantly, the absence of CD39 disrupts endothelial-dependent relaxation, reduces acute flow-mediated dilation, and decreases coronary output [109].

CD39 and CD73 are pivotal in modulating neuronal activity and synaptic plasticity within the nervous system. The deletion of microglial CD39 results in decreased striatal eADO, enhanced neuronal PKA signaling, and exacerbated D1-agonist-induced seizures [110]. Kainate-induced convulsions in the mouse hippocampus trigger ATP release, which subsequently leads to the upregulation of CD73 and A2AR expression. The excessive activation of A2AR significantly accelerates neurodegeneration [111]. Inhibition of CD73 reduces fear memory and disrupts the A2AR-mediated regulation of long-term potentiation in the amygdala [112]. Microglia form a dynamic network of ocular adenosine metabolism through their interactions with neurons and retinal blood vessels, mediated by CD39 and CD73 [83]. This interaction induces neuronal suppression through adenosine signaling and facilitates physiological hyperemia, representing a crucial mechanism in cerebrovascular reactivity and neurovascular coupling [113].

CD39 and CD73 are crucial regulators of the balance between osteoclasts and osteoblasts via adenosine and its receptors. The activation of A1R promotes osteoclast differentiation and function, whereas the activation of A2AR inhibits these processes [114]. CD39, which is derived from gingiva-derived mesenchymal stem cells, facilitates osteogenesis by activating the Wnt/β-catenin pathway [115]. A deficiency in CD73 disrupts osteoblast differentiation and leads to osteopenia, while CD73 overexpression enhances osteogenic differentiation through adenosine/A2BR pathway [116]. Additionally, apoptotic T-cell vesicles enrich CD39 and CD73 on their membranes, thereby facilitating bone regeneration through the A2BR and PKA pathways [117].

CD73 may also play a significant role in adipose tissue metabolism. Endothelial CD73-generated adenosine is internalized by adipocytes, converted to AMP *via* ADK, and subsequently activates AMP-activated protein kinase (AMPK), which suppresses de novo lipogenic gene expression [14]. GPI-anchored CD73 is transferred from adiposomes to rat adipocytes, promoting lipid synthesis in adipose tissue through a paracrine mechanism [118]. Under lipogenic stimuli, rat adipocytes release GPI-anchored CD73 into microvesicles, which then deliver CD73 to cytoplasmic lipid droplets, thereby promoting esterification [119].

#### Stress response regulation

The expression of CD39 and CD73 is influenced by environmental conditions and metabolic factors, such as hypoxia, oxidative stress, and nutritional deprivation. Various transcriptional regulatory mechanisms, including specificity protein 1, HIF-1α, and TGF-β, govern the expression of CD39 and CD73 [120–123].

The expression and enzymatic activity of CD39 and CD73 are significantly upregulated to enhance the conversion of ATP to AMP and adenosine under conditions of tissue hypoxia. The transcription factor specificity protein 1 is involved in the transcriptional regulation of CD39 and CD73 [120]. Hypoxia increases CD39 expression on endothelial cells through specificity protein 1, underscoring CD39's pivotal role in protective responses to hypoxic conditions [124]. The hypoxia-driven transcriptional target, HIF-1α, mediates the transcriptional upregulation of both CD39 and CD73 under hypoxic conditions [121]. Acute hypoxia significantly induces HIF-1α and doubles CD73 expression on tumor cells after 24 h at 1% O_2_, while the expression and activity of ADK remain unchanged [125]. In epithelial cells, hypoxia inhibits ENT2 in a HIF-1α-dependent manner, leading to a reduction in eADO uptake, which enhances mucosal adenosine signaling and consequently alleviates hypoxia-associated intestinal inflammation [126]. HIF stabilization transcriptionally enhances eADO production and signaling. The accumulated eADO primarily activates adenosine receptors, particularly A2AR and A2BR, thereby promoting hypoxic adaptation and enhancing cellular survival [127]. In contrast, supplemental oxygen (60% O_2_) reduces hypoxia, leading to decreased stabilization of HIF-1α and downregulation of its downstream targets, including CD39, CD73, and COX-2 [128]. This oxygenation significantly reduces eADO levels and adenosine receptor (A2AR/A2BR) signaling. Simultaneously, hyperoxia increases the expression of MHC class I on the surfaces of tumor cells, thereby enhancing their susceptibility to T cell-mediated cytotoxicity.

Oxidative stress induces overproduction of ROS, resulting in oxidative damage in both DNA and mitochondria. Oxidative stress significantly suppresses CD39 and CD73 enzymatic activity, exacerbating inflammation, infection, and tissue injury [129]. In contrast, upregulation of CD39 and CD73 exerts a protective effect by generating adenosine, thereby mitigating oxidative stress and cellular damage [130]. Interestingly, the expression of CD39 and CD73 protects cells from H_2_O_2_-induced oxidative stress and cytotoxicity, which correlates with enhanced activation of key signaling molecules, including AKT, ERK1/2, and MAPK [131]. Elevated expression and activity of CD39 enhance mitochondrial metabolism by inducing a cAMP-dependent mitochondrial stress response in acute myeloid leukemia, suggesting that modulation of CD39 may play a critical role in cell survival and the management of oxidative stress [132]. RBM15-mediated m6A modification increases CD39 activity, accelerating ATP degradation and adenosine generation [133]. This process subsequently activates the AKT/ERK/GSK3β and AMPK pathways, thereby enhancing antioxidant defenses and improving resistance to ischemic stress.

Nutritional stress conditions significantly alter the expression levels of CD73. Under nutrient deprivation, CD73 expression is increased by TGF-β activation; however, this upregulation can be reversed by blocking the TGF-β receptors/SMAD3 signaling pathway [122]. Furthermore, CD39 activates the TGF-β/SMAD3 pathway while simultaneously being subject to negative feedback regulation by SMAD3 [123].

Thus, hypoxia, oxidative stress, and nutrient deprivation each activate distinct transcriptional programs that regulate CD39 and CD73, thereby influencing the conversion of ATP to adenosine. Furthermore, the modulation of CD39 and CD73 may serve as a viable strategy to protect tissues from metabolic dysfunction and oxidative injury.

## CD39/CD73 in disease pathogenesis

**Fig. 3:**
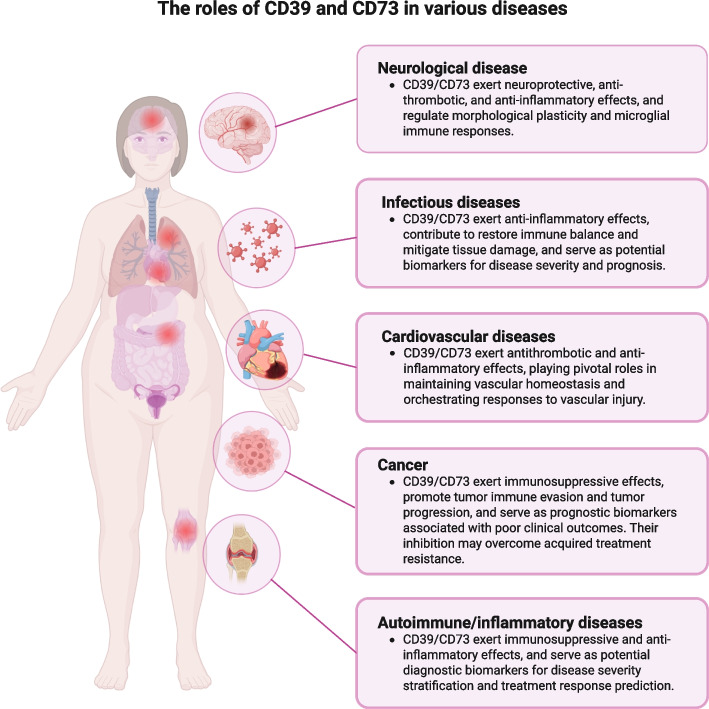
The roles of CD39 and CD73 in various diseases. CD39 and CD73 play critical roles in the production of eADO across various disease states, including cancer, autoimmune disorders, inflammatory diseases, cardiovascular diseases, infectious diseases, and neurological disorders. These enzymes exert immunosuppressive, antithrombotic, anti-inflammatory, and neuroprotective effects, serving also as potential biomarkers for disease pathogenesis, severity, and prognosis

Building upon their biological functions, CD39 and CD73 exert immunosuppressive, antithrombotic, anti-inflammatory, and neuroprotective effects in various diseases through their catalytic conversion to eADO. As illustrated in Fig. 3, CD39 and CD73 influence multiple pathological processes across diverse systems, including cancer, autoimmune and inflammatory diseases, cardiovascular disorders, infectious diseases, and neurological conditions. Notably, their expression and enzymatic activity not only impact disease progression but also position them as potential biomarkers for evaluating pathogenesis, monitoring disease severity, and predicting clinical outcomes. We will systematically delineate the roles of CD39 and CD73 across these varied disease states.

### Cancer

#### Tumor immune evasion and tumor progression

CD39 and CD73 exhibit a broad cellular distribution pattern in the TME, where they are prominently expressed among tumor cells, TILs, Tregs, and CAFs [27, 134–136]. They exert their enzymatic activity by catalyzing the conversion of ATP to eADO, thereby exerting immunosuppressive effects within TME and promoting tumor immune evasion and progression [33]. In addition to its established immunosuppressive function, CD39 demonstrates a range of additional capabilities, such as facilitating angiogenesis, enabling metabolic reprogramming, and contributing to intercellular communication networks [137]. This activity within the TME underscores the significant effects of CD39 and CD73 in promoting tumor growth. For instance, CD39 promotes tumor progression through its interaction with Annexin A2 and subsequent activation of the PI3K/AKT pathway [75]. CD73 has also been implicated in facilitating tumor progression through various signaling pathways, including the EGFR, MAPK, and PI3K/AKT pathways [138–140]. Additionally, CD73 expression is upregulated via MAPK/c-Jun signaling in ALK-rearranged, EGFR-mutant, and KRAS-mutant NSCLC cells [141]. In hepatocellular carcinoma, CD73 is significantly upregulated, leading to the binding of adenosine to A2AR, which stimulates AKT signaling through the Rap1/P110 cascade, thereby promoting tumor progression [140]. CD73 maintains cancer stem cell properties *via* AKT-mediated mechanisms, which reduce SOX9 degradation by inhibiting glycogen synthase kinase 3β and enhance its transcription *via* c-Myc [142]. CD73 promotes tumorigenesis by reprogramming lipid metabolism through a PR/PPARγ regulatory loop in mammary epithelial cells. In contrast, low expression or knockout of CD73 may increase tumor mutation burden (TMB) by disrupting this loop, thereby delaying the development of hormone receptor-negative tumors [143].

CD39 serves as a marker for exhausted, tumor-reactive CD8^+^ T cells [68]. Its elevated expression correlates with both M2 macrophage infiltration and CD8^+^ T cell exhaustion [144]. CD39 also acts as a pivotal regulator of CD4^+^ T cell maldifferentiation. Tumor cells sort CD39 into exosomes, which are then transferred to interacting T cells. This transfer induces ATP depletion and leads to the hyperactivation of AMP-activated kinase, ultimately driving the maldifferentiation of CD4^+^ T cells [145]. The densities of CD39^+^CD8^+^ TILs positively correlate with CD4^+^ T cells and B cells in the TME [25]. CD39^+^ tissue-resident memory CD8^+^ T cells exhibit clonal overlap across tumors and metastatic lymph nodes, playing a pivotal role in anti-tumor immunity [28].

CD73 plays a pivotal role in tumor progression by inhibiting the function of CD8^+^ T cells [139]. The expression of CD73 is inversely correlated with the levels of infiltration of CD8^+^ T cells and γδ T cells. High CD73 expression is associated with an increased TMB and elevated PD-L1 expression [146]. CD73 limits the differentiation and metabolic fitness of CD8^+^ T cells through the action of eADO, while CD73 deficiency enhances the cytotoxic potential of these cells, characterized by increased production of IFN-γ, TNF-α, and granzyme B, as well as elevated glucose uptake and heightened mitochondrial respiration, resulting in improved tumor growth inhibition [73]. Following CD73 inhibition, monocytes secrete increased levels of TNF-α, IL-2, and IFN-γ, thereby enhancing their phagocytic and cytotoxic abilities [147]. High expression of CD73 in melanoma is correlated with reduced intratumoral infiltration of NK cells and CD8^+^ T cells, thereby establishing an immunosuppressive TME [148]. Tumor-infiltrating CD73^+^ NK cells are associated with larger tumor sizes and express higher levels of immune checkpoints compared to CD73^−^ NK cells. These CD73^+^ NK cells undergo STAT3-mediated transcriptional reprogramming, which enhances IL-10 production, thereby suppressing the proliferation of CD4⁺ T cells and the secretion of IFN-γ [66].

CD39^hi^CD73^hi^ Tregs drive immunosuppression through the adenosine-A2AR pathway, suppressing CD8^+^ T cell proliferation and thereby facilitating tumor immune evasion [135]. Furthermore, elevated CD73 significantly contributes to immunosuppression in murine pancreatic ductal adenocarcinoma (PDAC) through paracrine signaling *via* the A2BR on CD8^+^ T cells [149]. CD73 upregulates C–C motif chemokine ligand 5, which facilitates the recruitment of Tregs and contributes to the immunosuppressive microenvironment; conversely, CD73 inhibition decreases tumor-infiltrating Tregs in pancreatic cancer [150]. In ovarian cancer, infiltration of γδ1 T cells is observed in both malignant ascites lymphocytes and TILs, with γδ1 T cells exhibiting notably elevated CD39 expression [151]. CD39^+^γδ Tregs have been identified in colorectal cancer, exhibiting superior immunosuppressive capabilities compared to CD4^+^ and CD8^+^ Tregs *via* the adenosine pathway [152]. In mouse KPC tumors, CD39/CD73 expressed by myeloid cells and CD73 expressed by tumors drive the polarization of myeloid cells towards M2-like macrophages, thereby accelerating tumor growth [153]. Additionally, adenosine generated by ovarian cancer cells attracts myeloid cells and promotes their differentiation into TAMs [79].

#### Metastasis and prognosis

Numerous preclinical studies have investigated the inhibition of CD39 and CD73 in mitigating tumor growth and metastasis [154]. The knockdown of CD73 gene significantly inhibits tumor growth, migration, and invasion, whereas the overexpression of CD73 promotes tumor proliferation in vitro and enhances tumor growth in vivo [140, 155]. CD73-mediated adenosine signaling modulates the RICS/RhoA pathway, inhibits LIM-kinase/cofilin phosphorylation, promotes epithelial-to-mesenchymal transition, and ultimately enhances tumor metastasis [155]. Numerous studies have demonstrated that elevated expression of CD39 and CD73 in tumor cells correlates with unfavorable clinical outcomes [26, 75, 136, 146, 156]. CD39 has the potential to act as a prognostic biomarker for various types of tumors, including head and neck squamous cell carcinoma, NSCLC, cholangiocarcinoma, gastric cancer, colorectal cancer, and ovarian cancer [26, 65, 75, 134, 136, 157]. Specifically, CD73 has been investigated as a prognostic biomarker for clinical outcomes in several solid tumors, including lung cancer, nasopharyngeal carcinoma, pancreatic cancer, esophageal carcinoma, thyroid carcinoma, gastric adenocarcinoma, and intrahepatic cholangiocarcinoma [35, 146, 156, 158–161].

Recently, the prognostic significance of CD73 in postoperative outcomes has been explored, identifying CD73 as an independent prognostic factor for disease-free survival (DFS) in patients with resected PDAC [162]. High CD73 expression not only serves as an independent prognostic marker associated with poor overall survival (OS), but CD73 inhibition also significantly suppresses the proliferation, migration, and invasion of gastric tumor cells [36]. In triple-negative breast cancer (TNBC), elevated CD73 expression on tumor cells correlates with poorer DFS and OS, as well as reduced tumor immune infiltration [163]. Patients with high CD73 expression (≥ 50%) on the tumor cells showed improved progression-free survival (PFS) and OS, particularly in EGFR-mutant NSCLC patients receiving immune checkpoint inhibitors (ICIs) targeting programmed cell death protein 1 (PD-1) and its ligand PD-L1 [164]. A study assessing the prognostic significance of CD73 in resected colorectal cancer with liver metastasis demonstrated that high intratumoral CD73 expression correlates with poorer patient outcomes, independent of clinicopathological variables. In contrast, soluble CD73 showed a weaker correlation with prognosis; however, patients with elevated soluble CD73 levels also experienced shorter disease-specific survival [165]. Furthermore, elevated CD73 expression in colon adenocarcinoma is associated with an increased risk of distant metastasis and a negative response to adjuvant chemotherapy [166]. Interactions between plasmacytoid DCs and multiple myeloma cells induce CD73 expression, and this elevated expression is significantly correlated with poor OS in multiple myeloma [78]. CD73 may also function as a prognostic marker for hematological malignancies [167].

Elevated CD39 expression is significantly associated with aggressive clinicopathological features and serves as an independent prognostic marker for poor outcomes in cholangiocarcinoma [75]. In high-grade serous ovarian carcinoma, the expression of CD39 and CD73 is primarily localized to CAFs and correlates with poor prognosis [136]. The elevated expression of CD39 and CD73 in the tumor stroma significantly correlates with reduced 5-year recurrence-free survival in early untreated NSCLC patients [26]. Importantly, the proportion of CD39^+^CD8^+^ T cells in TNBC patients is positively correlated with improved OS, indicating a prognostic association that extends to other CD39^+^ immune cell populations [168]. Elevated frequencies of CD39^hi^CD8^+^ TILs in gastric cancer serve as an independent prognostic factor for inferior OS [134]. Additionally, the frequency of CD39^+^ γδ Tregs is significantly positively correlated with adverse clinical outcomes [157].

#### Therapeutic resistance

Both congenital and acquired resistance to anti-tumor therapies is common, ultimately resulting in tumor recurrence and therapeutic failure. Consequently, it is crucial to investigate the mechanisms underlying both types of resistance in order to refine existing combination treatment strategies. Increasing evidence indicates that CD39 and CD73 are pivotal in the acquired resistance to cancer therapies.

Elevated CD39 activity drives cytarabine resistance in acute myeloid leukemia by activating a cAMP-dependent mitochondrial stress response, which enhances mitochondrial function and biogenesis. Conversely, the inhibition of CD39 prevents this metabolic reprogramming and significantly enhances the cytotoxicity of cytarabine [132]. In PDAC, increased CD73 expression promotes gemcitabine resistance through its intracellular interaction with major vault protein, which activates SRC-AKT signaling. Inhibition of CD73 restores sensitivity to gemcitabine, indicating that targeting CD73 could be a viable strategy to overcome gemcitabine resistance [169]. High levels of CD73 expression in biliary cancer correlate with aggressive biological behavior and resistance to standard chemotherapy, while CD73 inhibition increases sensitivity to cisplatin/gemcitabine treatment in biliary cancer [170]. CD73 acts as a downstream target of Notch1 and is upregulated by the overexpression of the Notch1 intracellular domain, promoting cisplatin resistance in TNBC cells; however, deletion of CD73 restores cisplatin sensitivity [171]. In NSCLC, tumor-derived TGF-β significantly enhances CD39 and CD73 expression on MDSCs, which subsequently suppress T cell and NK cell activity while shielding tumor cells from chemotherapy-induced cytotoxicity through the actions of CD39 and CD73 [23].

The cell-specific expression of CD39 in macrophages and CD73 in hepatocellular carcinoma cells synergistically enhances the ATP-adenosine pathway, leading to the generation of immunosuppressive adenosine that impairs CD8^+^ T cell function and promotes resistance to anti-PD-1 therapy. Notably, the inhibition of CD39 in macrophages restores the sensitivity to anti-PD-1 therapy in hepatocellular carcinoma [172]. Additionally, elevated CD39 expression on CD8^+^ T cells serves as a predictor of clinical response to ICIs, such as pembrolizumab, in TNBC [168]. Furthermore, increased CD39^+^ tumor-resident memory cells are associated with improved recurrence-free survival in stage III melanoma patients undergoing adjuvant immunotherapy, suggesting that this subset may function as a predictive biomarker for response to anti-PD-1 therapy [173]. Exosomal CD73 derived from the serum of melanoma patients generates immunosuppressive adenosine, thereby impairing T cell function. Elevated levels of exosomal CD73 in treatment non-responders indicate a correlation between exosomal CD73 expression and poor response to anti-PD-1 antibodies [174]. Moreover, the analysis of soluble CD73 enzymatic activity in stage IV metastatic melanoma patients receiving nivolumab therapy highlights its potential utility as a predictive biomarker for treatment response [175].

The expression of CD73 is associated with the response to BRAF inhibitors in melanoma. Melanoma cells that develop resistance to dabrafenib show increased CD73 expression, including higher levels of soluble CD73 released into the extracellular environment. This suggests that CD73 plays a significant role in acquired resistance to targeted therapy [176]. In cells resistant to first-generation EGFR tyrosine kinase inhibitors (EGFR-TKIs), the inhibition of CD73 results in increased cell cycle arrest and apoptosis, effectively overcoming acquired resistance [177]. Notably, elevated CD39 expression in CD8^+^ TILs was observed following radiotherapy. Further inhibition of CD39 not only diminished the exhausted phenotype of these CD8^+^ TILs post-radiotherapy but also synergistically enhanced their infiltration, proliferation, and cytokine production. This indicates that targeting CD39 may represent a promising strategy to mitigate radiotherapy resistance by alleviating the exhaustion of CD8^+^ TILs [178].

These findings demonstrate that CD39 and CD73 play pivotal roles in tumor immune evasion, progression, and metastasis through multifaceted mechanisms. As prognostic biomarkers across various cancer types, they facilitate biomarker-driven patient stratification. Furthermore, CD39 and CD73 are promising therapeutic targets, and their inhibition may help overcome acquired treatment resistance. Additional clinical validation is necessary to advance targeted therapeutic strategies.

### Autoimmune and inflammatory diseases

#### Immune regulation

CD39 and CD73 convert ATP into the immunosuppressive molecule eADO, playing critical roles in immune regulation across a variety of diseases, including rheumatoid arthritis, lupus, allergic airway diseases, inflammatory digestive tract disorders, and alcohol-related liver disease [29, 179–182]. On one hand, the expression levels of CD39 and CD73 are influenced by cytokine production and T cell activation [179]. On the other hand, CD39 and CD73 are pivotal in modulating T cell cytokine production.

In Crohn's disease, there is a significant enrichment of activated CD39^+^ and CD39^+^PD-1^+^ CD8^+^ T cell subsets, which express multiple exhaustion markers [183]. This is accompanied by a decrease in immunoregulatory CD39^+^ γδ T cells and an influx of immature CD39^-^ γδ T cells into the epithelium. Notably, early depletion of γδ T cells accelerates the onset of Crohn's disease-like ileitis [184]. In the context of lupus, CD39 serves as a marker for antibody-secreting cells in lupus-prone mice with stable profiles, thereby enhancing their characterization [185]. These cells play a crucial role in immune protection through their production of antibodies and cytokines. However, in systemic lupus erythematosus, CD73 on B cells remains inactive, resulting in reduced production of eADO and subsequent immune hyperactivation [186]. CD39^+^FOXP3^+^ Tregs accumulate at sites of inflammation, effectively suppressing pro-inflammatory cytokines such as IFN-γ and TNF when compared to CD39^−^ Tregs [187]. Furthermore, a CD39^+^CD9^+^ lung interstitial macrophage identified in neutrophil-dominant asthma mitigates IL-23/Th17-mediated neutrophilic inflammation through CD9-dependent neutrophil adhesion and CD39-dependent ATP hydrolysis [181]. Following liver injury, CD39 and CD73 expression is significantly upregulated on CD11b^+^Gr-1^+^ myeloid cells. Importantly, the CD39^+^CD73^+^Gr-1^high^CD11b^+^ subset secretes CD73-enriched extracellular vesicles that convert AMP, activate the intracellular cAMP pathway, and suppress CD4^+^ T-cell hyperactivation, thereby attenuating liver injury [188]. Furthermore, CD73 on endometrial regenerative cells orchestrates immunomodulation in concanavalin A-induced hepatitis by generating eADO, which suppresses CD4^+^ T cell activation and reduces liver damage; deletion of CD73 abrogates both hepatic protection and systemic immune regulation [189].

#### Anti-inflammatory effects

In addition to their roles in immune regulation, CD39 and CD73 modulate inflammatory processes through the enzymatic hydrolysis of ATP and the accumulation of eADO [190]. In various inflammatory disorders, CD39 and CD73 expressed by immune cells exert significant anti-inflammatory effects. These ectonucleotidases play a crucial role in protecting tissues from excessive inflammatory damage across a range of disorders.

CD73-deficient mice exhibit significantly exacerbated gastritis, accompanied by elevated levels of pro-inflammatory cytokines [29]. In chronic pancreatitis, CD73 knockout mice demonstrate markedly enhanced acinar-to-ductal metaplasia, indicative of persistent inflammation [191]. During pancreatitis, CD39 is overexpressed in vascular and adjacent tissues; however, CD39-deficient mice exhibit inflammatory responses characterized by significantly elevated levels of IFN-γ in both tissues and plasma, along with reduced fibrogenesis [192]. CD39 and CD73 are expressed on rheumatoid arthritis synoviocytes, facilitating the production of eADO and thereby exerting anti-inflammatory effects [193]. The anti-inflammatory role of CD73 is further underscored by studies in a mouse model, where CD73 deficiency results in increased levels of pro-inflammatory cytokines in the joints, heightened Th1 cell responses, and aggravated joint destruction [194]. CD39- and CD73-deficient mice demonstrate significant endothelial dysfunction and an increased release of neutrophil extracellular traps following lupus induction, underscoring the protective capacity of CD39 and CD73 [180]. Additionally, CD73 expression is markedly elevated in sensitized airways, while CD73-deficient mice exhibit reduced allergen-induced airway hyperreactivity due to decreased Th2 activation and diminished mast cell recruitment and degranulation [195]. Elevated levels of CD39^+^ plasma microparticles are observed in patients with liver injury, and mobilized CD39 attenuates vascular inflammation and promotes hepatic regeneration [196]. In a mouse model of alcohol-induced liver injury, CD73 overexpression mitigates liver damage, decreases lipid accumulation, and suppresses the secretion of pro-inflammatory cytokines through the inhibition of the TLR4/MyD88/NF-κB pathway [182].

#### Potential biomarker

In the context of autoimmune and inflammatory diseases, CD39 and CD73 are crucial for maintaining immune homeostasis and regulating inflammatory responses. Notably, the expression patterns and enzymatic activities of CD39 and CD73 on immune cells, as well as in biological fluids, are not only correlated with disease activity but also possess significant potential as predictors of therapeutic response.

The frequencies of CD39^+^CD73^+^ Tregs are diminished in rheumatoid arthritis, and this reduction exhibits an inverse correlation with disease activity scores [179]. Furthermore, elevated CD39 expression on B cells is inversely correlated with rheumatoid factor levels and disease activity. In treatment-responsive patients with rheumatoid arthritis, CD39 expression on B cells increases post-treatment, whereas non-responders demonstrate a decrease in expression [197]. The frequency of CD39^+^CD73^+^ B cells is lower in systemic lupus erythematosus compared to healthy controls. This frequency shows positive correlations with prothrombin time and IgM levels, while exhibiting negative correlations with IL-6, IFN-α, C-reactive protein, anti-double-stranded DNA antibodies, and the Disease Activity Index [198]. Serum CD39 levels in allergic rhinitis are reduced, particularly in moderate-to-severe cases, indicating that serum CD39 may serve as a promising diagnostic biomarker with significant potential for stratifying disease severity in allergic rhinitis [199]. The enzymatic activity of soluble CD73 at hospital admission is a predictor for the development of severe pancreatitis, with both the activity and protein concentration of soluble CD73, as well as CD73 mRNA levels, declining as disease severity increases [200].

These findings indicate that CD39 and CD73 act as master regulators of immune homeostasis, mediating anti-inflammatory effects across a variety of autoimmune and inflammatory diseases. Furthermore, CD39 and CD73 are recognized as promising biomarkers for diagnostic stratification, monitoring disease activity, and predicting treatment responses in these disorders. Therefore, utilizing CD39 and CD73 for patient stratification and investigating their therapeutic potential represents a viable strategy.

### Cardiovascular diseases

Intravascular ATP, ADP, and their metabolic product, eADO, are crucial regulators of vascular physiology, significantly influencing vascular tone and hemodynamic stability. Variations in extracellular nucleotide levels activate signaling pathways that result in platelet activation, as well as the activation and recruitment of leukocytes and endothelial cells [31]. CD39 and CD73 facilitate the hydrolysis of ATP, producing the anti-inflammatory and antithrombotic mediator adenosine, which is essential for maintaining vascular homeostasis and coordinating responses to vascular injury. The roles of CD39 and CD73 have been observed in conditions such as atherosclerosis, myocardial infarction, and cardiac arrest.

In atherosclerotic mice, CD39 expression is prominently observed in stable regions characterized by atheroprotective flow, while it is significantly reduced in areas prone to atherosclerosis, which are marked by disturbed flow [201]. Serum levels of ATP, ADP, and CD73 are significantly elevated in conditions of atherosclerosis [202]. The genetic deletion of CD73 in apolipoprotein E-deficient mice results in an approximately 50% increase in the formation of atherosclerotic lesions, likely due to the loss of CD73's inhibitory regulation on resident macrophages and T cells [203]. Persistent T cell receptor signaling within the vascular microenvironment fosters a CD8^+^ T cell phenotype characterized by the upregulation of CD39 and a decrease in cytokine production [204]. Notably, reduced CD39 expression on Tregs correlates with an exacerbated atherosclerotic burden in both human and mouse models [205].

Furthermore, CD39 plays a protective role in myocardial ischemia/reperfusion injury, as evidenced by significantly larger infarct sizes observed in CD39-deficient mice following ischemic challenges [206]. Activated Tregs contribute to protection against ischemia/reperfusion injury through a CD39-dependent mechanism, which mitigates cardiomyocyte apoptosis, activates AKT/ERK signaling pathways, and limits neutrophil infiltration [207]. The expression of CD39 induced by TGF-β1 establishes a negative feedback loop that reduces fibroblast activation; in contrast, CD39 deficiency leads to enhanced collagen deposition, decreased elastin expression, and aggravated diastolic dysfunction [208]. During myocardial infarction, CD73 expressed on T cells is crucial for modulating cardiac wound healing through the enhanced hydrolysis of ATP and the activation of adenosine-mediated A2AR/A2BR signaling [30]. Moreover, CD73-deficient mice subjected to ischemia/reperfusion demonstrate significantly reduced cardiac adenosine release, severe post-infarction cardiac dysfunction, increased infarct expansion, and widespread ventricular fibrosis [209]. Additionally, diminished CD73 levels on CD4^+^ T cells correlate with elevated serum markers of cardiac injury, including NT pro-BNP and myocardial enzymes [210].

In rodent models of post-cardiac arrest syndrome, alterations in immune cell populations reveal differential regulation of CD39 and CD73. Specifically, CD39 and CD73 are upregulated on CD11b^+^ myeloid cells and CD19^+^ B lymphocytes, while their expression is downregulated on CD3^+^ T lymphocytes [211]. Furthermore, plasma levels of CD39 and CD73 correlate with impaired myocardial perfusion reserve, indicating ectonucleotidase shedding from the vascular endothelium [212]. In cardiac arrest survivors, CD73 expressed on lymphocytes exerts a protective effect against inflammation by suppressing the activation of pro-inflammatory myeloid cells and enhancing the secretion of vascular endothelial growth factor (VEGF) [213]. Additionally, CD73 expressed on T cells demonstrates significant anti-inflammatory effects in heart failure induced by transverse aortic constriction, primarily through the suppression of cardiac fibrosis and the reduction of pro-inflammatory cytokine production, mediated by adenosine and A2A receptor activation [214]. CD73-deficient mice exhibit exacerbated myocardial fibrosis and cardiomyocyte hypertrophy, indicating that CD73 plays a crucial protective role against congestive heart failure, ventricular hypertrophy, and fibrosis [215].

These findings establish CD39 and CD73 as crucial regulators of cardiovascular homeostasis through the generation of adenosine, while also highlighting their role in modulating immune cell responses. The cardioprotective functions of CD39 and CD73 observed in cardiovascular diseases emphasize the therapeutic potential of targeting this pathway.

### Infectious diseases

Infection triggers inflammatory responses through the release of extracellular ATP, which is subsequently degraded by CD39 and CD73 to produce the anti-inflammatory mediator eADO. The critical balance between pro-inflammatory ATP and anti-inflammatory eADO ultimately determines the outcomes of infection and inflammation [216]. Increasing evidence indicates that CD39 and CD73 serve dual roles—both protective and pathogenic—in various infectious diseases, including coronavirus disease 2019 (COVID-19), human immunodeficiency virus (HIV) infection, and sepsis.

In COVID-19 patients, dysregulated nucleotide metabolism is characterized by elevated expression of CD39 and CD73 in total leukocytes, altered hydrolysis of platelet ATP, ADP, and AMP in severe cases, increased extracellular ATP levels, and upregulated pro-inflammatory cytokines such as IL-2, IL-6, IL-10, and IL-17. These findings indicate significant alterations in purinergic signaling and immune responses during the pathogenesis of COVID-19 [32]. Furthermore, elevated plasma levels of soluble CD39 in COVID-19 patients are associated with prolonged hospitalization and serve as independent predictors of admission to the intensive care unit, suggesting that soluble CD39 may act as a potential biomarker for disease severity in COVID-19 [217]. COVID-19 patients also exhibit significantly increased levels of Tregs, particularly a marked rise in CD39^+^ Tregs and CD39^+^CD4^+^ T cell subsets. These immunological alterations are accompanied by downregulation of CD73 expression and a notable reduction in serum adenosine levels, indicating their potential contribution to immune dysfunction and disease progression through the elevation of CD39^+^ Tregs and the reduction of adenosine [218]. Additionally, the mild-recovered group shows increased co-expression of CD39^+^CD73^+^ Tregs, with CD39^+^CD73^+^ Tregs being significantly more prevalent among volunteers experiencing myalgia [219].

In HIV infection, CD39 expression is significantly elevated on Tregs, and the expansion of these CD39^+^ Tregs is associated with increased immune activation and a decline in CD4^+^ T cell counts [220]. CD39^+^ Tregs suppress IL-2 production in CD4^+^ T cells through the CD39/A2AR/cAMP pathway, and a high frequency of CD39^+^ Tregs correlates with poor clinical outcomes in HIV infection [221]. Although the frequencies of CD73^+^ T cells are reduced across T cell subsets in HIV infection, CD73^+^CD8^+^ T cells exhibit both diminished cytokine production (including TNF-α, IFN-γ, and IL-2) and upregulated expression of homing receptors (CCR7 and α4β7 integrin), indicating that these cells possess enhanced migratory capacity [222]. Notably, HIV infection alters the expression patterns of CD39 and CD73 on γδ T cells, which correlates with immune activation and disease progression [70].

In murine models of sepsis, the upregulation of CD39 and CD73 expression plays a crucial role in mitigating inflammation, organ damage, immune cell apoptosis, and bacterial load [223, 224]. CD73 knockout mice demonstrate increased thymic apoptosis, lung injury, kidney injury, and mortality during sepsis, which correlate with elevated bacterial loads and heightened levels of pro-inflammatory cytokines and chemokines [224]. In macrophages, CD39 modulates the P2X7-mediated pro-inflammatory response by scavenging ATP and generating eADO, which activates A2AR. This activation not only limits systemic inflammation but also reduces liver injury induced by sepsis [225]. The expansion of CD39^high^ B cells, particularly CD39^high^ plasmablasts, during murine sepsis, along with the resultant accumulation of adenosine, impairs the bactericidal function of macrophages while enhancing IL-10 production through A2AR signaling [226]. Furthermore, analysis of CD39^+^ Tregs in pediatric sepsis reveals a negative correlation with the Phoenix sepsis score. Patients exhibiting CD39^+^ Treg levels below the critical threshold of 12.45% face a significantly heightened risk of mortality, suggesting that CD39^+^ Tregs may serve as promising prognostic biomarkers [227].

These findings highlight the dual role of CD39 and CD73 in infectious diseases. On one hand, they can provide protective effects by resolving excessive inflammation and mitigating organ damage, as seen in sepsis. On the other hand, they may also contribute to a pathogenic role by fostering immune dysfunction and facilitating disease progression, as evidenced in COVID-19 and HIV infections.

### Neurological diseases

An increasing body of evidence suggests that CD39 and CD73 are involved in various mechanisms underlying neurological diseases, such as cerebrovascular disorders and epilepsy spectrum disorders. These ectonucleotidases play a crucial role in modulating purinergic signaling through the generation of eADO, which subsequently influences inflammation, neurotransmission, and neuroprotection.

In CD39-transgenic mice subjected to cerebral ischemia, CD39 overexpression attenuates cerebral ischemic injury, as evidenced by decreased leukocyte infiltration, reduced infarct sizes, and improved neurological deficits [228]. CD39-deficient mice demonstrate significantly larger cerebral infarcts and impaired post-ischemic cerebral perfusion [229]. In ischemic stroke, eADO generated by CD39 and CD73 exerts anti-inflammatory and neuroprotective effects through the activation of ARs. The anti-inflammatory effect is mediated by the activation of A2AR on infiltrating immune cells, while neuroprotection is attributed to A1R activation, which suppresses glutamatergic excitotoxicity [230]. Lymphocytes from ischemic stroke patients exhibit elevated A2AR expression and enhanced affinity, indicating a compensatory mechanism to counteract post-ischemic inflammation and leukocyte infiltration. Conversely, the observed decrease in CD73^+^ cells within CD4^+^ and CD8^+^ T lymphocyte subsets likely contributes to sustained inflammation despite A2AR upregulation [231]. In ischemic stroke patients, both the frequency and immunosuppressive capacity of Tregs are significantly impaired, with the most profound decrease observed in the active CD39^+^FoxP3^+^ Treg subset [232].

In mice subjected to kainate-induced convulsions, an elevation in ATP release is observed, along with increased levels of CD73 and A2AR in hippocampal synapses and astrocytes. These changes are associated with impaired memory performance and long-term potentiation, underscoring the critical role of the CD73-A2AR pathway in synaptic dysfunction and neurodegeneration following convulsions [111]. In the context of the epileptic brain, neuronal hyperexcitability triggers microglial activation and upregulates CD39 expression. Subsequently, these activated microglia exert a feedback inhibitory effect on neuronal hyperexcitability in a manner dependent on the enzymatic activity of CD39 [233].

These studies demonstrate the anti-inflammatory and neuroprotective roles of CD39 and CD73 in neuroinflammation and neuronal homeostasis across various neurological conditions. Consequently, CD39 and CD73 are emerging as promising therapeutic targets for the treatment of neurological disorders.

## Therapeutic targeting of CD39/CD73

**Fig. 4:**
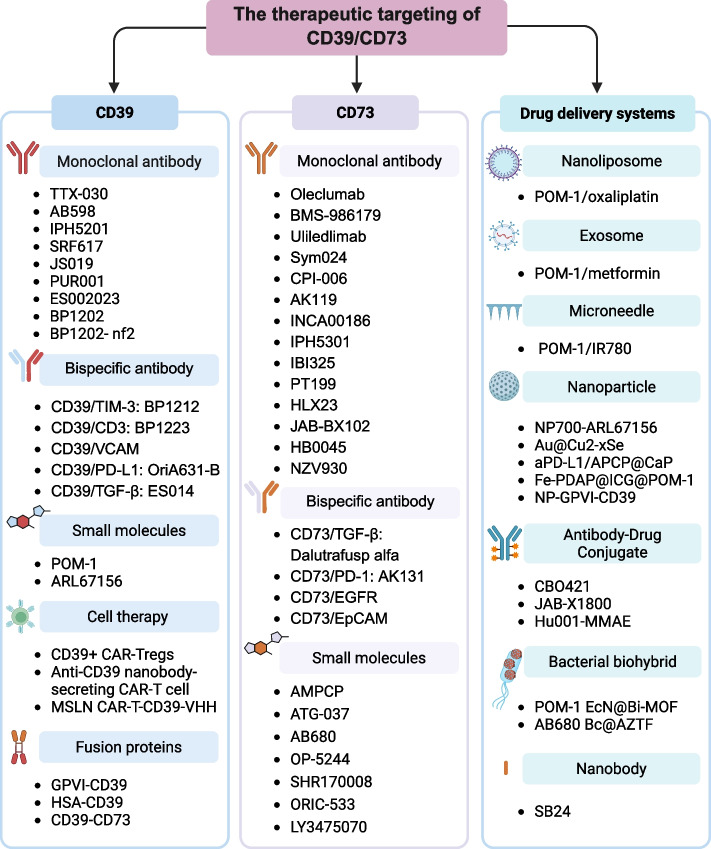
The therapeutic targeting of CD39/CD73. The advancement in targeting CD39/CD73 has involved the development of monoclonal antibodies, bispecific antibodies, and small-molecule inhibitors. Moreover, innovative biologics, such as engineered cell therapies and fusion proteins, have expanded the therapeutic options available for this pathway. Additionally, novel drug delivery systems—including nanoliposomes, exosomes, microneedles, nanoparticles, antibody–drug conjugates, bacterial biohybrids, and nanobodies—have been developed to enhance targeting and efficacy

Given the roles of CD39 and CD73 in mediating immunosuppressive, antithrombotic, anti-inflammatory, and neuroprotective effects across various diseases, targeting these proteins has emerged as a promising therapeutic strategy. As illustrated in Fig. 4, significant efforts have been devoted to developing agents that target CD39 and CD73, including small-molecule inhibitors, monoclonal antibodies, bispecific antibodies, fusion proteins, and cell therapies. To address pharmacokinetic challenges, innovative delivery systems have been developed, such as nanoliposomes, exosomes, microneedles, nanobodies, antibody–drug conjugates, and bacterial biohybrids. The following sections will systematically review current strategies for the design and blockade of CD39/CD73 inhibitors, along with key preclinical and clinical advancements.

### CD39-targeted therapies

Table 1 provides a detailed overview of the clinical trials involving CD39 inhibitors registered in the United States National Clinical Trials Registry. Of these trials, 64.71% are currently in phase I, while 35.29% have progressed to phase II. The primary therapeutic focus of these studies is on advanced solid tumors, with several agents, including TTX-030, IPH5201, SRF617, ES014, and TIN816 having advanced to phase II development for this indication.

**Table 1:** CD39 inhibitors in clinical trials. (Source: https://clinicaltrials.gov/)

Drug	Indication	Phase	NCT number	Treatment
**Anti-CD39 monoclonal antibody**				
TTX-030	Advanced solid tumors	Phase I	NCT04306900	+ Pembrolizumab/Budigalimab ± Chemotherapy
	Solid tumor, lymphoma	Phase I	NCT03884556	Monotherapy, + Pembrolizumab/Chemotherapy
	Pancreatic cancer	Phase II	NCT06119217	+ Chemotherapy ± Budigalimab
AB598	Advanced solid tumors	Phase I	NCT05891171	Monotherapy, + Zimberelimab + FOLFOX
IPH5201	Advanced solid tumors	Phase I	NCT04261075	Monotherapy, + Durvalumab ± Oleclumab
	NSCLC	Phase II	NCT05742607	+ Durvalumab + Chemotherapy
SRF617	Advanced solid tumors	Phase I	NCT04336098	Monotherapy, + Pembrolizumab, + Gemcitabine + Albumin-bound paclitaxel ± Pembrolizumab
	Prostate cancer	Phase II	NCT05177770	+ Etrumadenant + Zimberelimab
JS019	Advanced solid tumors	Phase I	NCT05508373	Monotherapy
	Advanced solid tumors or lymphomas	Phase I	NCT05374226	Monotherapy
PUR001	Advanced solid tumors	Phase I	NCT05234853	Monotherapy
ES002023	Advanced solid tumors	Phase I	NCT05075564	Monotherapy
**CD39-targeting bispecific antibodies**				
ES014 (CD39/TGF-β)	Advanced solid tumors	Phase I	NCT05381935	Monotherapy
			NCT05717348	Monotherapy
		Phase II	NCT06543056	Monotherapy
**Recombinant human CD39 enzyme**				
TIN816	Sepsis-associated acute kidney injury	Phase II	NCT05507437	Monotherapy
			NCT05996835	Monotherapy

#### Anti-CD39 antibodies

TTX-030 is a first-in-class, fully human anti-CD39 monoclonal antibody that functions as an uncompetitive allosteric inhibitor [234]. It suppresses CD39 ATPase activity, thereby enhancing anti-tumor immunity by decreasing immunosuppressive adenosine and preserving extracellular ATP levels. Epitope mapping has confirmed that TTX-030 binds to a region on the N-terminal lobe of CD39 (E142-Y159), which is located distal to the enzymatic active site. Furthermore, TTX-030 has been investigated in combination with budigalimab (an anti-PD-1 antibody) and FOLFOX chemotherapy as a first-line treatment for patients with locally advanced or metastatic gastric or gastroesophageal junction cancer. According to the 2022 American Association for Cancer Research (AACR) meeting, the most commonly reported adverse events (AEs) included nausea, decreased neutrophil count, decreased appetite, diarrhea, and fatigue. Notably, 11% of patients experienced Grade 3/4 AEs associated with TTX-030. Additionally, 21 patients achieved a partial response (PR), 2 achieved a complete response (CR), 12 had stable disease (SD), and 3 had progressive disease (PD) [235]. At the 2024 European Society for Medical Oncology (ESMO) congress, TTX-030 combined with nab-paclitaxel and gemcitabine, with or without budigalimab, demonstrated favorable safety and promising efficacy as a first-line treatment for PDAC (NCT04306900), particularly in patients with high expression of human leukocyte antigen-DQ. This finding has prompted an ongoing phase II trial (NCT06119217) for further validation [236].

AB598 is an Fc-silent, humanized anti-CD39 monoclonal antibody distinguished by its sub-nanomolar binding affinity and potent enzymatic inhibitory activity [38]. It effectively inhibits CD39 enzymatic activity, resulting in elevated intratumoral ATP levels and significant tumor growth control in both MOLP8 murine xenograft models and cynomolgus monkeys, thereby supporting its clinical development [237]. In the context of combination therapy that incorporates immunogenic cell death (ICD)-inducing chemotherapy along with AB598, the ICD-inducing agents promote the release of extracellular ATP, while AB598 preserves extracellular ATP levels by inhibiting hydrolysis mediated by CD39. This combination therapy not only enhances DC maturation but also stimulates T cell activation, thereby amplifying anti-tumor immunity [38]. Currently, AB598 is under evaluation in a phase I clinical trial (NCT05891171) as both a monotherapy and in combination with Zimberelimab and FOLFOX for the treatment of advanced solid tumors.

IPH5201, another human anti-CD39 monoclonal antibody, specifically targets both membrane-bound and soluble forms of CD39, effectively inhibiting the enzymatic conversion of ATP to eADO [238]. It demonstrates enhanced anti-tumor activity when combined with oxaliplatin in human CD39 knock-in mice [238]. Currently, IPH5201 is being evaluated in both a phase I (NCT04261075) and a phase II (NCT05742607) clinical trial.

SRF617, a fully human anti-CD39 antibody, has been shown to reduce ATPase activity and CD39 expression while simultaneously enhancing CD8^+^ T cell infiltration within the TME, ultimately leading to tumor growth inhibition [239]. Currently, it is under investigation in a phase I clinical trial (NCT04336098) targeting advanced solid tumors. At the 2021 ESMO congress, SRF617 demonstrated favorable tolerability, with fatigue, nausea, and constipation identified as the most common treatment-emergent adverse events (TEAEs). Additionally, the recommended phase II dose for SRF617 has been established at 1400 mg administered every two weeks [240].

BP1202 is a novel humanized anti-CD39 antibody that exhibits a high affinity for recombinant CD39 protein. It is capable of inhibiting CD39 enzyme activity and enhancing anti-tumor immunity by specifically targeting CD39 on intratumoral and exhausted T cells [241]. BP1202-NF2, a glycosylated variant of the anti-CD39 antibody, depletes CD39^high^ Tregs through antibody-dependent cellular cytotoxicity, while simultaneously enhancing antigen-specific cytotoxic T lymphocyte responses, thereby promoting anti-tumor immunity [242]. Additionally, several bispecific antibodies targeting CD39 are currently under development. BP1212, a bispecific antibody that targets both CD39 and T cell immunoglobulin and mucin domain-containing protein 3 (TIM-3), effectively blocks immunosuppressive pathways by inhibiting TIM-3 signaling and adenosine generation [243]. BP1223, another bispecific antibody that targets CD39 and CD3, demonstrates potent anti-tumor activity in acute myeloid leukemia through effective T cell activation and the induction of bystander cytotoxicity [244]. Furthermore, a CD39/vascular cell adhesion molecule 1 (VCAM1) bispecific antibody, significantly mitigates lipopolysaccharide-induced cytokine storms, hypothermia, myocardial stress, and hypoxic drive in septic mice, showcasing its strong anti-inflammatory and cardiorespiratory protective effects [245]. In murine models of ischemic stroke, the anti-VCAM1-CD39 antibody exhibits antithrombotic and anti-inflammatory properties by delivering recombinant CD39 to activated penumbral endothelium, which enhances cerebral perfusion and maintains blood–brain barrier integrity [246]. OriA631-B (a CD39/PD-L1 bispecific antibody) displays potent anti-tumor efficacy in syngeneic MC38 and Hepa1-6 models through the dual blockade of CD39-mediated immunosuppression and PD-L1-dependent T cell exhaustion, while also demonstrating a favorable safety profile [247]. ES014 (a CD39/TGF-β bispecific antibody) counteracts adenosine-induced immune tolerance and TGF-β-mediated Treg differentiation, protecting effector T cells from activation-induced cell death, and exhibits a favorable safety profile in cynomolgus monkeys [248]. ES014 is currently undergoing phase I and phase II clinical trials for solid tumors (NCT05717348, NCT06543056).

#### Small molecule inhibitors of CD39

Sodium polyoxotungstate (POM-1), an inorganic metal cluster, serves as a nonspecific small-molecule inhibitor of CD39. In addition to its potent inhibition of CD39, POM-1 and other polyoxometalates are capable of inhibiting additional members of the NTPDase family, as well as ENPP1 and alkaline phosphatases [249]. POM-1 enhances T-cell tumor infiltration and inflammatory responses in a humanized mouse model of Epstein-Barr virus (EBV)-driven lymphomagenesis [250]. In bladder cancer models, CD39 inhibition with POM-1 alters the TME by increasing the presence of NK cells, conventional type 1 DCs, and CD8^+^ T cells, while reducing Tregs [251]. This treatment approach restricts tumor progression and extends survival. In a mouse glioma model, the combination of POM-1 and Adriamycin enhances immune cell infiltration and suppresses tumor growth, indicating that CD39 plays a crucial role in the immune escape of glioma stem cells [252].

ARL67156 has demonstrated therapeutic efficacy in mitigating the progression of aortic stenosis. In a rat model of calcific aortic valve disease, treatment with ARL67156 significantly reduces both apoptosis and mineralization in aortic valve tissues [253]. Furthermore, in a mouse model of Hutchinson-Gilford progeria syndrome, the combined administration of ARL67156, ATP, and levamisole enhances the synthesis of pyrophosphate while inhibiting its degradation, effectively preventing vascular calcification and extending lifespan [254].

#### Other therapeutic strategies

Advanced drug delivery systems utilizing micro/nanotechnology approaches have been developed to enhance therapeutic efficacy. A liposomal nanoplatform that co-delivers POM-1 and the ICD inducer oxaliplatin effectively conceals POM-1 to minimize toxicity, facilitates tumor-penetrant release, and reduces immunosuppression, thereby significantly inhibiting tumor growth and inducing immune memory [255]. Cancer cell-derived exosomes co-loaded with POM-1 and metformin enhance pro-inflammatory extracellular ATP levels, which activate the P2X7-NLRP3 inflammasome, induce macrophage pyroptosis, boost DC capacity, and promote T/NK cell-mediated cytotoxicity, thereby driving synergistic anti-tumor immunity [256]. A dissolving microneedle patch has been designed for the co-delivery of POM-1 and the ICD inducer IR780, aiming to simultaneously induce ICD and reduce eADO, thereby enhancing anti-tumor therapy [257]. ROS-generating nanoparticles (NP700) loaded with ARL67156 have been developed to simultaneously induce ATP release and inhibit its conversion to eADO, thereby enhancing tumor-specific T cell responses and promoting tumor regression in mouse models [258]. Concurrently, a bacterial-based biohybrid composed of bismuth and ellagic acid delivers POM-1, enhancing radiotherapy-induced ICD while inhibiting the ATP-to-eADO conversion pathway, resulting in a durable anti-tumor immune response [259]. A photothermal nanomedicine, designated as Fe-PDAP@ICG@POM-1, was engineered through the electrostatic adsorption of POM-1, iron-doped polydiaminopyridine, and indocyanine green. This novel photothermal nanomedicine facilitates dual-directional immunometabolic regulation by inhibiting the ATP-adenosine pathway, which serves to reverse the tumor immunosuppressive microenvironment [260]. Furthermore, SB24, a novel anti-CD39 nanobody derived from cDNA-immunized alpacas, effectively inhibits both soluble and membrane-bound CD39. The dimerization of SB24 via Fc fusion significantly enhances its blockade efficacy and addresses the penetration limitations commonly associated with traditional anti-CD39 antibodies [261].

The engineering of CD39 fusion proteins represents an emerging therapeutic strategy, exemplified by glycoprotein VI-Fc and CD39 fusion proteins, human serum albumin (HSA)-CD39 fusion proteins, and bifunctional CD39-CD73 fusion proteins. Although current antithrombotic therapies are limited by the risk of bleeding, nanoparticle-based delivery of dimeric glycoprotein VI-Fc-CD39 fusion protein (NP-GPVI-CD39) specifically inhibits platelet aggregation and thrombus formation [262]. The developed human serum albumin-CD39 fusion protein effectively degrades pro-inflammatory ATP and ADP into AMP, exhibiting excellent hemocompatibility and reduced cell adhesion when coated on medical devices [263]. Intriguingly, engineered bifunctional CD39-CD73 fusion proteins scavenge pro-inflammatory ATP while generating anti-inflammatory eADO, thereby suppressing platelet activation in response to exogenous agonists [264]. These properties position CD39 fusion proteins as promising candidates for the treatment of thrombosis and inflammation.

CD39-targeting cell therapy represents a promising strategy in the field of immunotherapy. In the context of chimeric antigen receptor (CAR)-Treg therapy for type 1 diabetes, CD39^+^ CAR-Tregs exhibit lower granzyme B expression and reduced cytotoxicity [265]. A single-epitope anti-CD39 nanobody effectively inhibits CD39, thereby enhancing T-cell proliferation and function, while also demonstrating anti-tumor activity in mouse models. The incorporation of this nanobody into CAR-T cells significantly improves therapeutic efficacy against ovarian cancer [266]. Furthermore, mesothelin CAR-T cells exhibit notable upregulation of CD39 upon activation by ovarian cancer cells. Consequently, MSLN CAR-T-CD39-VHH, engineered to secrete the anti-CD39 antibody OriA631, displays enhanced anti-tumor efficacy alongside reduced antibody-associated toxicity [267]. The exploration of autologous cell therapy has also advanced, particularly with PD-1^+^CD39^+^ selected TILs, which demonstrate multifunctional superiority through increased cytokine secretion, multi-cancer reactivity, and potent autologous tumor cytotoxicity [268].

### CD73-targeted therapies

Table 2 provides a detailed overview of the clinical trials involving CD73 inhibitors. The developmental landscape for CD73 inhibitors exhibits greater diversity compared to that of CD39: 38.36% are in phase I, 23.29% are in phase I/II, 32.88% are in phase II, 1.37% are in phase II/III, and 4.10% are in phase III. Non-small cell lung cancer (NSCLC) has emerged as the predominant focus among investigations into advanced solid tumors, with oleclumab identified as the most extensively studied candidate. Furthermore, oleclumab, AB680, and CPI-006 have progressed to phase III clinical evaluation (NCT05221840, NCT06608927, NCT04734873).

**Table 2:** CD73 inhibitors in clinical trials. (Source: https://clinicaltrials.gov/)

Drug	Indication	Phase	NCT number	Treatment
Anti-CD73 monoclonal antibody				
Oleclumab	Advanced solid tumors	Phase I	NCT03736473	Monotherapy
			NCT02503774	Monotherapy, + Durvalumab
			NCT02740985	+ Durvalumab + AZD4635
	Ovarian Cancer	Phase II	NCT03267589	+ Durvalumab
	NSCLC	Phase I	NCT03801902	+ Durvalumab + Radiation therapy
			NCT03819465	+ Durvalumab ± Chemotherapy
		Phase I/II	NCT03381274	+ Osimertinib/AZD4635
		Phase II	NCT03822351	+ Durvalumab
			NCT03794544	+ Durvalumab
			NCT05061550	+ Durvalumab + Platinum doublet chemotherapy
			NCT03334617	+ Durvalumab
			NCT03833440	+ Durvalumab
		Phase III	NCT05221840	+ Durvalumab
	NSCLC or renal cell cancer	Phase II	NCT04262375	+ Durvalumab
	Pancreatic cancer	Phase I/II	NCT03611556	+ Chemotherapy ± Durvalumab
		Phase II	NCT04940286	+ Gemcitabine + Nab-paclitaxel + Durvalumab
	Prostate cancer	Phase II	NCT04089553	+ AZD4635
	TNBC	Phase I/II	NCT03616886	+ Paclitaxel + Carboplatin + Durvalumab
			NCT03742102	+ Paclitaxel + Durvalumab
	Luminal B breast cancer	Phase II	NCT03875573	+ Chemotherapy + Radiotherapy ± Durvalumab
	Bladder cancer	Phase I	NCT03773666	+ Durvalumab
	Colorectal cancer	Phase I/II	NCT04068610	+ FOLFOX + Bevacizumab + Durvalumab
		Phase II	NCT04145193	+ Durvalumab + mFOLFOX6
	PDAC	Phase II	NCT06060405	+ Durvalumab
	PDAC, NSCLC, squamous cell carcinoma of head and neck	Phase II	NCT04262388	+ Durvalumab
	Multiple sarcoma	Phase II	NCT04668300	+ Durvalumab
BMS-986179	Solid tumors	Phase I/II	NCT02754141	Monotherapy, + Nivolumab/rHuPH20
Uliledlimab	Solid tumors	Phase I	NCT03835949	+ Atezolizumab
	Advanced solid tumors	Phase I/II	NCT04322006	Monotherapy, + Toripalimab
	Advanced or metastatic solid tumors	Phase II	NCT05001347	+ Atezolizumab
	NSCLC	Phase II	NCT07005336	+ Sintilimab + Chemotherapy
		Phase II/III	NCT06984588	+ Toripalimab
Sym024	Advanced solid tumors	Phase I	NCT04672434	Monotherapy, + Sym021
	NSCLC	Phase I/II	NCT06162572	+ Cemiplimab
AK119	Advanced solid tumors	Phase I	NCT05173792	Monotherapy
			NCT04572152	+ AK104
		Phase I/II	NCT05559541	+ AK104
			NCT05689853	+ AK112
	NSCLC	Phase I/II	NCT05636267	+ AK112 ± Chemotherapy
	Colorectal cancer	Phase I/II	NCT05846867	+ AK112 ± FOLFIRI
	COVID-19	Phase I	NCT04516564	Monotherapy
JAB-BX102	Advanced solid tumors	Phase I/II	NCT05174585	Monotherapy, + Pembrolizumab
CPI-006	Advanced solid tumors	Phase I	NCT03454451	Monotherapy, + Ciforadenant/Pembrolizumab
	COVID-19	Phase I	NCT04464395	Monotherapy
		Phase III	NCT04734873	+ Standard of Care
INCA00186	Advanced solid tumors	Phase I	NCT04989387	Monotherapy, + INCB106385 ± Retifanlimab
IPH5301	Advanced solid tumors	Phase I	NCT05143970	Monotherapy, + Trastuzumab + Paclitaxel
IBI325	Advanced solid tumors	Phase I	NCT05119998	Monotherapy, + Sintilimab
			NCT05246995	+ Sintilimab
NZV930	Advanced solid tumors	Phase I	NCT03549000	Monotherapy, + PDR001 ± NIR178
			NCT04237649	+ KAZ954
HLX23	Advanced solid tumors	Phase I	NCT04797468	Monotherapy
PT199	Advanced solid tumors	Phase I	NCT05431270	Monotherapy, + Tislelizumab
HB0045	Advanced solid tumors	Phase I/II	NCT06056323	Monotherapy
CD73-targeting bispecific antibodies				
Dalutrafusp alfa (CD73/TGF-β)	Advanced solid tumors	Phase I	NCT03954704	Monotherapy, + mFOLFOX6
	Advanced pancreatic cancer	Phase II	NCT05632328	+ Botensilimab ± Chemotherapy
	Colorectal cancer	Phase II	NCT06300463	+ Botensilimab + Balstilimab
AK131 (CD73/PD-1)	Advanced solid tumors	Phase I	NCT06166888	Monotherapy
Small-molecule CD73 inhibitor				
AB680	Pancreatic cancer	Phase I	NCT04104672	+ Zimberelimab + Nab-paclitaxel + Gemcitabine
		Phase I/II	NCT05688215	+ Zimberelimab + mFOLFIRINOX
		Phase II	NCT06048484	+ Zimberelimab + Etrumadenant
		Phase III	NCT06608927	+ Nab-paclitaxel + Gemcitabine
	Advanced upper gastrointestinal tract malignancies	Phase II	NCT05329766	+ Zimberelimab
	NSCLC	Phase II	NCT05676931	+ Zimberelimab ± Docetaxel, + Zimberelimab + Platinum-Based Doublet ± Domvanalimab
	Advanced biliary tract cancers	Phase II	NCT06048133	+ Zimberelimab + Gemcitabine + Cisplatin
	Colorectal cancer	Phase I/II	NCT04660812	+ Etrumadenant + Zimberelimab
	Prostate Cancer	Phase I/II	NCT04381832	+ Etrumadenant ± Zimberelimab
		Phase II	NCT05915442	+ Etrumadenant + Zimberelimab + Ablative radiation
ATG-037	Advanced solid tumors	Phase I	NCT05205109	Monotherapy, + Pembrolizumab
ORIC-533	Multiple myeloma	Phase I	NCT05227144	Monotherapy
LY3475070	Advanced solid tumors	Phase I	NCT04148937	Monotherapy, + Pembrolizumab

#### Anti-CD73 antibodies

BMS-986179 is a humanized immunoglobulin G2 (IgG2) monoclonal antibody that exhibits high-affinity binding to CD73. The primary results of the Phase I study on BMS-986179 (NCT02754141) were presented at the 2018 AACR meeting. Treatment-related adverse events (TRAEs) were reported in 58% of patients, with grade 3 TRAEs occurring in 15%. Among the 59 patients enrolled, 7 patients with pancreatic cancer, head and neck cancer, anal cancer, prostate cancer, and renal cancer, achieved a confirmed PR, while 10 exhibited SD [269]. Given the safety concerns and limited efficacy gains associated with the addition of nivolumab, the further development of BMS-986179 appears to have been halted after Phase I.

Another significant discovery is oleclumab (also known as MEDI9447), a humanized IgG1 monoclonal antibody that inhibits CD73 [270]. In mice with NSCLC cells harboring EGFR mutations, the combination of the anti-PD-L1 antibody durvalumab and oleclumab significantly decreased tumor volume [271]. Given the acceptable AEs and favorable outcomes observed in clinical trials, oleclumab may represent a promising anti-CD73 monoclonal antibody. However, a Hook Effect has been noted following high-dose administration of oleclumab. The Hook Effect, also known as the loss of enzymatic inhibition, is a phenomenon observed in enzymatic inhibition assays. At high concentrations of the inhibitor, the inhibitory effect on enzymatic activity initially increases in a dose-dependent manner but decreases after reaching a peak. In the context of CD73-mediated AMP hydrolysis, the hook effect occurs when the CD73 inhibitor is present in molar excess relative to the CD73 homodimer. This results in reduced binding efficiency or a complete loss of CD73 enzymatic inhibition, primarily due to factors such as monovalent binding, steric hindrance, or competition for limited binding sites [272]. Insights derived from the hook effect observed with oleclumab have underscored that binding geometry and stoichiometry are critical determinants of CD73 inhibition efficacy. These findings have catalyzed the rational design of anti-CD73 monoclonal antibodies that employ alternative binding modes—such as intra-dimer engagement, non-competitive inhibition, or multi-epitope targeting—to mitigate or entirely abolish the Hook Effect.

Compared to oleclumab, IPH5301 is more effective in inhibiting AMP hydrolysis and the enzymatic activity of CD73 [238]. It primarily interacts with the N-terminal region of CD73 dimers, which are formed in a 1:1 stoichiometry. This interaction induces an intermediate state in CD73, rendering it incapable of hydrolyzing AMP. Uliledlimab (also known as TJ004309) completely inhibits CD73 through a non-competitive intra-dimer binding mode by binding to a unique C-terminal epitope. This mechanism avoids the hook effect and preserves potent enzymatic inhibition [273]. PT199 completely inhibits both membrane-bound and soluble shed CD73 by binding to a unique epitope, with no observable hook effects. It exhibits high affinity for both open and closed conformations, effectively inhibiting CD73 by preventing the transition from the open to the closed state [274]. Similarly, IBI325 demonstrates inhibitory effects on both soluble and cell-bound forms of CD73, also without any Hook Effects [275].

Sym024 (also referred to as S095024) binds to a distinct epitope on the CD73 enzyme, situated contralateral to the catalytic center. This binding bridges both monomers of the enzyme and interacts with a 1:1 stoichiometry, resulting in profound inhibition of CD73 activity and effectively suppressing tumor growth in vivo [276, 277]. Furthermore, a novel therapeutic strategy involving an anti-CD73 antibody cocktail, HB0045, which is a 1:1 mixture of humanized IgG1 antibodies HB0038 and HB0039, has shown the capability to stabilize CD73 in a partially open conformation. This stabilization occurs as the antibodies bind to different sites on the enzyme through the Fabs of HB0038 and HB0039, without eliciting a Hook Effect [278].

Notably, CPI-006 activates B cells and upregulates CD69 expression, leading to a reduction of circulating CD73-positive B cells and an increased CD4:CD8 ratio [279]. AK119 has been shown to induce B cell proliferation and enhance the expression levels of CD69 and CD83, showcasing superior bioactivity in B cell activation compared to oleclumab or CPI-006 [280]. Beyond cancer therapy, both CPI-006 and AK119 are being evaluated for the treatment of COVID-19 (NCT04516564, NCT04464395, NCT04734873).

Several bispecific antibodies targeting CD73, in conjunction with other targets, are currently under development. Dalutrafusp alfa, a bispecific antibody that targets both CD73 and TGF-β (also known as GS-1423/AGEN1423), is being investigated for its ability to inhibit the production of eADO mediated by CD73 and to counteract the immunosuppressive effects of TGF-β [281]. The results of the GS-US-505–5452 study, which evaluated dalutrafusp alfa monotherapy in patients with advanced solid tumors (NCT03954704), have been published. The most frequently reported TRAEs included fatigue, nausea, vomiting, and diarrhea. Notably, grade 3 or 4 TRAEs were observed in 42.9% of patients. Among the 17 patients evaluated, a PR was noted in 4.8% (specifically in endometrial cancer), SD was observed in 33.3% (across ovarian cancer, liposarcoma, non-small cell lung cancer, renal cell carcinoma, and colon cancer), and PD was documented in 42.9% (including cases of colon cancer, adrenal cancer, pancreatic cancer, appendiceal cancer, ovarian cancer, and endometrial cancer) [282]. Building on these findings, a phase II study (NCT05632328) is currently underway [283].

AK131, a bispecific antibody targeting CD73 and PD-1, is derived from AK119 and AK105. It enhances the activation of both B and T cells, thereby inhibiting tumor growth in mouse models [284]. In preclinical studies, AK131 demonstrates a more potent immunostimulatory effect compared to anti-PD-1 or anti-PD-L1 antibodies, whether used alone or in combination with CD73 antibodies [285]. Another bifunctional PD-L1/CD73 inhibitor, CC-5, significantly inhibits tumor growth, and increases the populations of CD3^+^ and CD8^+^ T cells [285]. Additionally, a bispecific antibody targeting both CD73 and EGFR enhances the infiltration of CD8^+^ T cells into tumors, activates macrophages, and sensitizes tumors to the cytotoxic effects of chemotherapy [286]. Furthermore, a CD73/epithelial cell adhesion molecule (EpCAM) bispecific antibody reduces proliferation in ovarian cancer cells and enhances sensitivity to chemotherapy [287]. Collectively, bispecific antibodies targeting CD73 exhibit synergistic effects by concurrently engaging multiple mechanisms, thereby enhancing anti-tumor immune responses. As research progresses, these bispecific antibodies hold considerable promise for patients.

#### Small molecule inhibitors of CD73

The first small molecule inhibitor of CD73, derived from ADP, identified in 1970, was 5’-(α, β-methylene-adenosine) diphosphate (AMPCP, also known as AOPCP) [288]. AMPCP engages with the active site of CD73 through a combination of polar, ionic, and hydrophobic interactions. Notably, this includes bifurcated hydrogen bonds formed by the ribose moiety with residues D506 and N390, a face-to-face π-stacking interaction between the adenine heterocycle and residues F417 and F500, and an ionic interaction between the bisphosphonate group and the di-Zn catalytic domain [289].

The development of small molecule inhibitors targeting CD73 presents several challenges, including inadequate metabolic stability, poor tumor-specific delivery, and the reversible nature of inhibition. For example, AMPCP inhibits not only the AMPase activity of CD73 but also the nucleotide phosphodiesterase activities of ENPP1 and ENPP3 [290]. However, it demonstrates significantly reduced stability in human blood plasma, resulting in its degradation [291]. Phosphonate-based CD73 inhibitors are characterized by poor membrane permeability and low oral bioavailability, which are commonly associated with nucleotide phosphonates [292]. Therefore, the development of novel small-molecule inhibitors for CD73 is of paramount importance.

The advancement of small molecule inhibitors of CD73 primarily utilizes AMPCP as the lead compound for structural optimization. This includes the malonic acid analogue ATG-037 (also known as CB708), the diphosphonic acid analogue AB680 (also known as quemliclustat), and the methylenephosphonic acid analogue OP-5244. AB680 features a central azaindazole core derived from 4,6-dichloro-1H-pyrazolo[3,4-b]pyridine 3 [293], as detailed in studies of the crystal structure of human CD73 and the structure–activity relationships involving N6-substituted AMPCP derivatives [294]. Unlike AMPCP, the π-stacking interaction of AB680 is influenced by the nucleobase, which alters the interaction between F417 and F500, resulting in a face-to-face π-interaction between F417 and the fluorophenyl substituent [289]. SHR170008 was developed by modifying the linkage between the ribose and α-phosphate of AMPCP. Enhancements in potency and metabolic stability were achieved through alterations to the adenine structure of AMPCP [295]. Additionally, non-nucleoside inhibitors of CD73 have been identified from anthraquinone, sulfa, benzosulfonamides, (diaryl)methyl phosphonic acid derivatives, and benzotriazole analogues [292, 296–299].

PSB-12489, along with other small-molecule CD73 inhibitors exhibiting sub-nanomolar potency, was developed using AMPCP as a lead compound [300]. The design of PSB-12489 involved structural modifications of AMPCP, including a chloro substitution at the C2-position and the introduction of a benzyl group and a methyl group at the N6-position of the adenine nucleobase. PSB-12489 demonstrates metabolic stability, high selectivity, and outstanding potency, while also mitigating the risk of forming adenosine receptor-activating compounds, thus preventing potential serious side effects. The AMPase activity of CD73 is inhibited by PSB-12489, leading to a significant reduction in melanoma cell invasion in a transwell system [122].

AB680 is a highly potent, selective, and reversible inhibitor of CD73, marking it as the first small molecule inhibitor of CD73 to advance to clinical-stage development. It demonstrates significant effects on human CD8^+^ T cells, with a half-maximal inhibitory concentration (IC50) of less than 0.01 nM [301]. In mouse models of PDAC, AB680 reduces tumor growth while simultaneously increasing the populations of DCs, activated CD8^+^ T cells, and macrophages [302]. Furthermore, AB680 decreases the expression of eADO-regulated NR4A genes and enhances tumor inflammation in metastatic PDAC [303]. AB680 have revealed prolonged half-lives and extremely low clearance rates in both rodent and non-rodent species [304]. Currently, AB680 is in phase III clinical development (NCT06608927).

In contrast to AB680, which necessitates intravenous administration, ATG-037 and ORIC-533 provide the benefit of oral bioavailability. In comparison to oleclumab and Hu101-28, ATG-037 not only restores T cell function but also reverses AMP-dependent T cell suppression, exhibiting a lower IC50 value of 0.36 nM [305]. Furthermore, ATG-037 achieves complete inhibition of the CD73 enzyme without exhibiting a Hook Effect [306]. Currently, ATG-037 is under evaluation in Phase I clinical trials for advanced solid tumors (NCT05205109). Preliminary results presented at the 2025 American Society of Clinical Oncology (ASCO) meeting indicated that among 43 enrolled patients receiving monotherapy, 21 patients experienced SD, resulting in a disease control rate (DCR) of 49%. TRAEs occurred in 24 patients (56%), with the most frequently observed TRAEs being of grade 1–2 severity [307]. ORIC-533 effectively inhibits eADO production from AMP, significantly reducing the viability of multiple myeloma cells and restoring their lysis [308]. It demonstrates superior efficacy compared to oleclumab in enhancing CD8^+^ T cell proliferation under immunosuppressive conditions, and it surpasses small molecule inhibitors targeting the adenosine receptor and CD73 [309]. In autologous co-culture models using bone marrow mononuclear cells from multiple myeloma patients, ORIC-533 has been shown to elicit anti-multiple myeloma immunity, resulting in the destruction of tumor cells. Furthermore, it enhances cytotoxicity mediated by NK cells and induces ICD [310]. ORIC-533 has been assessed in a Phase I clinical trial (NCT05227144) involving patients with relapsed or refractory multiple myeloma. According to findings presented at the 65th American Society of Hematology (ASH) meeting, the plasma half-life of ORIC-533 is approximately 24 h. Additionally, an increase in circulating NK and CD8^+^ T cells was noted following the first cycle of treatment. Grade 3 TRAEs were reported in five patients, with fatigue being the most prevalent [311].

#### Other therapeutic strategies

Various drug delivery and nanotherapeutic techniques have been developed to enhance drug delivery and facilitate CD73 inhibition. One such approach is the drug Fc-conjugate CBO421, which combines a small molecule CD73 inhibitor with the human IgG1 Fc to silence immune responses. This conjugate shows complete inhibition of CD73 and enhances the internalization of the CD73 receptor compared to oleclumab [312]. Another promising candidate, JAB-X1800, is a CD73-targeted STING immunostimulatory antibody–drug conjugate (ADC), which demonstrates good tolerance in animal models while maintaining stability in plasma [313]. Additionally, a monomethyl auristatin E (MMAE)-conjugated CD73-ADC, known as Hu001-MMAE, protects T effector cells, activates DCs, exhibits cytotoxicity against tumor cells with high CD73 expression, and enhances the tumor immune response [314]. Additionally, an engineered biohybrid (Bc@AZTF) is created by grafting prebiotic bacteria with the ICD inducer AZTF, encapsulating AB680 within a core carrier made from ZIF-90, and coating it with an iron-polyphenol layer. This biohybrid plays a crucial role in activating ICD therapy and inhibiting the production of the immunosuppressive adenosine [315]. AmGd-NPs, nanoscale coordination particles comprised of the self-assembly of AMPCP and high-Z metal gadolinium (Gd), are capable of releasing AMPCP to inhibit CD73 enzyme activity [316]. Au@Cu2-xSe nanoparticles function as CD73 inhibitors by releasing copper ions that chelate with disulfides, leading to the formation of a cytotoxic complex that inhibits CD73 expression, reduces eADO-driven immunosuppression, and enhances T cell recruitment [317]. Calcium phosphate nanoparticles have been engineered for the co-delivery of the CD73 inhibitor APCP and the anti-PD-L1 antibody. These nanoparticles exhibit pH-responsive drug release properties, an exceptional drug-loading capacity, and a synergistic anti-tumor effect, while significantly minimizing systemic toxicity and immune-related adverse effects (irAEs) [318].

### Combination therapies

Current evidence indicates that monotherapy with CD39/CD73 inhibitors exhibits limited efficacy, highlighting the need for therapeutic optimization and the development of rational combination strategies. As a result, an increasing number of clinical trials are concentrating on combination therapies, which include adenosine receptor antagonists, targeted therapies, immunotherapy, and chemotherapy.

Preclinical studies have demonstrated that the combination of CD39/CD73 inhibitors with A2AR antagonists improves anti-tumor efficacy and reduces tumor burden [319, 320]. When used in combination with the A2AR/A2BR antagonist INCB106385, INCA00186 significantly inhibits the growth of human MDA-MB-231 breast cancer cells compared to monotherapies [321]. The combination of the A2AR antagonist AZD4635 with oleclumab has indicated anti-tumor activity and a tolerable safety profile in the treatment of prostate cancer (NCT04089553) [322]. Preliminary findings from a study investigating the combination of CPI-006 and the A2AR antagonist ciforadenant suggest an increased response rate without a significant rise in TRAEs compared to CPI-006 monotherapy [279]. In a murine model of multiple myeloma, the combined blockade using POM-1, AZD4635, and an anti-CD73 antibody significantly enhances the activation of immune cells, increases IFN-γ levels, and reduces tumor burden [320]. Furthermore, the simultaneous application of CD73 inhibitors with both A2AR and A2BR antagonists has demonstrated improved anti-tumor efficacy [49].

The combination of CD39/CD73 inhibitors with targeted therapies has been studied in both preclinical and clinical settings. Notably, the incorporation of a CD39 inhibitor into a treatment regimen that combines a CD20 inhibitor and a CD47 inhibitor results in a significant enhancement of survival in a model of disseminated aggressive B-cell lymphoma [323]. In nodal B-cell lymphoma, an anti-CD20 bispecific antibody combined with either anti-CD39 or anti-CD73 antibodies exhibits a synergistic effect, leading to increased cytokine secretion, T-cell proliferation, and tumor cell cytotoxicity [324]. Another promising strategy to enhance therapeutic efficacy involves the combination of CD73 inhibitors with EGFR-TKIs. A Phase Ib/II study (NCT03381274) evaluating the combination of oleclumab and the EGFR-TKI osimertinib in patients with advanced NSCLC harboring EGFR mutations was reported at the 2021 AACR meeting [325] and has been recently published [326]. The results demonstrated an objective response rate (ORR) of 11.8% at a dose of osimertinib 80 mg orally once daily, in combination with oleclumab 3000 mg. Additionally, the median PFS was reported to be 7.4 months longer than that observed with osimertinib alone, while the median OS was noted at 24.8 months. Grade 3 TRAEs occurred in 14.3% of patients, indicating that the combination of oleclumab and osimertinib is associated with acceptable tolerability [326].

Numerous preclinical studies have explored the efficacy of CD39 inhibitors in combination with ICIs or chemotherapy. The anti-CD39 monoclonal antibody demonstrates significant antimetastatic efficacy across various tumor models, outperforming POM-1, CD73 inhibitors, and A2A receptor antagonists. Furthermore, combination regimens involving anti-PD-1, IL-15/IL-2, or A2A receptor antagonists synergistically enhance metastatic control [327]. In a murine glioma model, the combination of POM-1 and Adriamycin markedly enhances the infiltration of immune cells into tumors, decreases tumor burden, and extends survival [278]. In breast cancer models, the combination of ARL67156, AMPCP, and doxycycline enhances anti-tumor immunity by inhibiting the polarization of TAMs and preventing extracellular ATP depletion induced by both tumor cells and infiltrating immune cells [328].

In contrast, multiple clinical trials have explored the therapeutic potential of CD73 inhibitors in combination with ICIs or chemotherapy. According to the 2024 ASCO Gastrointestinal Cancers Symposium, the results of the ARC-8 study (NCT04104672) in patients with metastatic PDAC, revealed that 85% of patients experienced grade ≥ 3 TRAEs, with the most common being decreased neutrophil count and anemia. In cohort A2, receiving AB680 plus nab-paclitaxel and gemcitabine, the median PFS was 8.8 months, and the median survival follow-up was 21.1 months. Conversely, in the cohort receiving AB680 with nab-paclitaxel, gemcitabine, and zimberelimab, the median PFS was 5.4 months, with a median survival follow-up of 20.3 months [329]. The results from the 2025 ASCO meeting demonstrated promising activity of ATG-037 combined with pembrolizumab in patients exhibiting acquired resistance to ICIs. Among 28 treated patients, 7 achieved confirmed PR (5 melanoma, 2 NSCLC) with an ORR of 25% [307]. These findings suggest that this combination may represent a viable therapeutic option for NSCLC and melanoma.

According to information presented at the 2023 ASCO meeting, a phase I/II study (NCT04322006) evaluated uliledlimab, both as a monotherapy and in combination with the anti-PD-1 antibody toripalimab for the treatment of advanced solid tumors. The preliminary cut-off for defining CD73^High^ was established at 40% of immune or tumor cells exhibiting ≥ 1 + staining intensity. The ORR of 50% in CD73High patients was significantly higher than the ORR of 14.8% observed in CD73^Low^ patients [158]. These findings suggest the association between high CD73 expression and enhanced clinical response to therapy. Additionally, combinations of CD73 inhibitors with PD-L1 inhibitors are expected to markedly improve anti-tumor efficacy. According to the D6070C00001 study (NCT02503774), in the expansion cohorts involving combination therapy with oleclumab and durvalumab for NSCLC, PDAC, and colorectal cancer, the corresponding ORRs were 9.5%, 4.8%, and 2.4%, respectively, while the 6-month PFS rates were 16.0%, 13.2%, and 5.4%, respectively [330]. The exploratory analysis of baseline tumor samples, which evaluates CD73 expression in relation to the best overall response, suggests that elevated CD73 expression may be associated with clinical benefits in microsatellite-stable colorectal cancer and PDAC [330].

The results of the Hudson study (NCT03334617) were recently published. In the cohort receiving both durvalumab and oleclumab, one PR was reported, accounting for 1.8% of patients with advanced NSCLC. The most frequently observed TRAEs included pruritus, fatigue, and diarrhea, with a grade ≥ 3 TRAE rate of 17.5% [331]. According to the report from the 2024 ASCO meeting, the addition of oleclumab and monalizumab to durvalumab in the phase II COAST study (NCT03822351) involving patients with stage III unresectable NSCLC, resulted in increased ORR (23.9% vs. 35.0% vs. 40.3%), prolonged median PFS (7.3 months vs. 21.1 months vs. 19.8 months), and improved 2-year OS rates (61.5% vs. 76.8% vs. 72.1%), importantly, the safety profiles across all treatment arms were comparable [332]. Additionally, the results of another study (NCT03611556) focusing on metastatic pancreatic cancer reported grade ≥ 3 TRAEs at rates of 67.7%, 73.7%, and 77.1% in expansion cohorts receiving chemotherapy, oleclumab with chemotherapy, and oleclumab with durvalumab plus chemotherapy, respectively; the ORRs were 29.0%, 21.1%, and 32.9% [333]. Based on extensive clinical trial data, the combination therapy of oleclumab and durvalumab in patients with NSCLC warrants further evaluation in the phase III study (NCT05221840) [334].

### Challenges and limitations

An increasing number of preclinical and clinical trials targeting CD39 and CD73 are being initiated. Some trials have demonstrated promising safety and efficacy profiles for various CD39/CD73 inhibitors in cancer treatment, with multiple candidates currently at different stages of development. Recent advancements in engineered CD39/CD73 inhibitors, particularly when integrated with sophisticated drug delivery systems, are emerging as a prominent area of research, despite being in the early phases of development. Notably, the efficacy of CD73 inhibitors is markedly enhanced when administered in conjunction with ICIs. Although these findings expand the therapeutic potential of CD39 and CD73 inhibitors, their development faces several significant challenges.

The efficacy of these inhibitors may be limited when administered as monotherapy due to broad expression of CD39 and CD73. Additionally, potential risks associated with off-target effects and irAEs may arise from the reactivation of T cells induced by CD73 [286]. Furthermore, the heterogeneous distribution of CD73, A2AR, and A2BR within the TME influences the function of CD73 and eADO [335]. However, some CD39 and CD73 inhibitors have demonstrated limited efficacy, attributed to factors such as incomplete enzyme inhibition, the hook effect, and the slow action of large molecules in vivo. Certain small molecule inhibitors exhibit less favorable pharmacokinetic properties, including poor oral absorption due to a strong acidic moiety, ionic characteristics, and high overall polarity. Additionally, high plasma protein binding limits their therapeutic efficacy [289, 336]. Following structural optimization, small molecule inhibitors can be administered orally with high bioavailability. These optimized inhibitors demonstrate good stability in plasma and provide superior exposure compared to macromolecules within the TME [337]. However, despite these advantages, the sustained anti-tumor activities of these small molecule inhibitors in vivo may be affected by their ability to effectively penetrate physiological barriers and their high metabolic rate.

Based on these findings, future strategies for developing CD39/CD73 inhibitors may include: (1) the design of novel small molecules, (2) structural optimization, (3) the creation of bispecific antibodies targeting different antigens, (4) the enhancement of drug delivery systems to ensure high drug loading efficiency while minimizing toxicity, (5) the implementation of cocktail therapies aimed at multiple epitopes, (6) improvements in tumor selectivity, (7) the exploration of biomarkers for patient stratification, and (8) the combination with other therapeutic modalities. To optimize combination therapies based on CD39/CD73 inhibitors, several critical factors must be addressed: effective management of TRAEs to maintain therapeutic feasibility, refinement of treatment protocols to enhance therapeutic effects, development of biomarker-guided dosing strategies, and overcoming mechanisms of treatment resistance. Addressing these challenges will be essential for the advancement of these promising combination therapies.

## Conclusion and prospects

Recent advances in the study of CD39 and CD73 have significantly enhanced our understanding of their biological functions, which include structural features, enzymatic properties, immunomodulatory effects, non-immune roles, and molecular interactions. CD39 and CD73 function as ectonucleotidases in the sequential hydrolysis of ATP to ADP and subsequently to AMP, ultimately leading to the production of eADO. These enzymes play a crucial role in various physiological processes, including vascular homeostasis, regulation of thrombosis, modulation of neural activity, neurovascular coupling, bone remodeling, and the metabolism of adipose tissue.

Targeting CD39 and CD73 presents significant clinical potential for various pathological conditions, including cancer, autoimmune disorders, inflammatory diseases, cardiovascular diseases, infectious diseases, neurological disorders, respiratory diseases, metabolic disorders, and skin diseases. To date, a growing range of inhibitors targeting CD39 and CD73 includes small molecules, monoclonal antibodies, and bispecific antibodies [33, 154]. There has been an increase in clinical trials investigating CD39 and CD73 inhibitors, not only as monotherapies but also in combination with adenosine receptor antagonists, targeted therapies, immunotherapy, and chemotherapy.

Preclinical and clinical studies have demonstrated the therapeutic potential of targeting CD39 and CD73 in cancer. However, a more comprehensive understanding of their mechanistic roles in disease pathogenesis is still required, and their therapeutic potential in other diseases remains relatively underexplored. Future research should emphasize the broader ectoenzyme network that regulates ATP-adenosine metabolism, as this may enhance the therapeutic efficacy of interventions targeting CD39 and CD73. Additionally, CD39 and CD73 should be evaluated as predictive and prognostic biomarkers. Therefore, further investigation is required to determine the optimal therapies involving CD39/CD73 inhibitors and the most effective dosing schedules to fully leverage their advantages.

## Data Availability

Not applicable. The data and materials supporting our conclusion of this review are included within the manuscript.

## Abbreviations

AACR: American Association for Cancer Research
ACRs: ATPase-conserved regions
ADA: Adenosine deaminase
ADK: Adenosine kinase
ADP: Adenosine diphosphate
ADPR: ADP-ribose
AEs: Adverse events
AK: Adenylate kinase
AMP: Adenosine monophosphate
AMPCP: 5’-(α, β-Methylene-adenosine) diphosphate
AMPK: AMP-activated protein kinase
ASCO: American Society of Clinical Oncology
ASH: American Society of Hematology
ATP: Adenosine triphosphate
cADPR: Cyclic ADP-ribose
CAFs: Cancer-associated fibroblasts
cAMP: Cyclic AMP
CAR: Chimeric antigen receptor
CD39: Cluster of differentiation 39
CD73: Cluster of differentiation 73
cGAMP: Cyclic GMP-AMP
cGAS: Cyclic GMP-AMP synthase
Conx: Connexin
COVID-19: Coronavirus disease 2019
CR: Complete response
CNT: Concentrative nucleoside transporter
DCR: Disease control rate
DCs: Dendritic cells
DFS: Disease-free survival
eADO: Extracellular adenosine
EBV: Epstein-Barr virus
EGFR: Epidermal growth factor receptor
EpCAM: Epithelial cell adhesion molecule
ESMO: European Society for Medical Oncology
ENPP1: Ectonucleotide pyrophosphatase/phosphodiesterase 1
ENT: Equilibrative nucleoside transporter
HCy: L-homocysteine
HIF-1α: Hypoxia-inducible factor 1 alpha
Gd: Gadolinium
GPI: Glycosylphosphatidylinositol
HIV: Human immunodeficiency virus
HSA: Human serum albumin
HYP: Hypoxanthine
ICD: Immunogenic cell death
ICIs: Immune checkpoint inhibitors
IFN: Interferon
IgG: Immunoglobulin G
INO: Inosine
IL: Interleukin
irAEs: Immune-related adverse effects
KRAS: Kirsten rat sarcoma viral oncogene homolog
MAPK: Mitogen-activated protein kinase
MDH: Malate dehydrogenase
MDSCs: Myeloid-derived suppressor cells
MMAE: Monomethyl auristatin E
mTOR: Mechanistic target of rapamycin
NAD: Nicotinamide adenine dinucleotide
NDPK: Nucleoside diphosphate kinases
NK: Natural killer
NMN: Nicotinamide mononucleotide
NO: Nitric oxide
NPPs: Nucleotide pyrophosphatase/phosphodiesterases
NR: Nicotinamide riboside
NLRP3: NLR family pyrin domain-containing protein 3
NRK1: Nicotinamide riboside kinase1
NSCLC: Non-small cell lung cancer
NTPDases: Nucleoside triphosphate diphosphohydrolases
ORR: Objective response rate
OS: Overall survival
Panx: Pannexin
PD: Progressive disease
PD-1: Programmed cell death protein 1
PDAC: Pancreatic ductal adenocarcinoma
PFS: Progression-free survival
PI3K/AKT: Phosphatidylinositol 3-kinase/protein kinase B
PKA: Protein kinase A
PNP: Purine nucleoside phosphorylase
PPi: Inorganic pyrophosphate
POM-1: Sodium polyoxotungstate
PR: Partial response
ROS: Reactive oxygen species
SAH: S-adenosyl-homocysteine
SAHH: S-adenosyl-homocysteine hydrolase
SD: Stable disease
STING: Stimulator of interferon genes
TAMs: Tumor-associated macrophages
TEAEs: Treatment-emergent adverse events
TGF-β: Transforming growth factor-beta
Th: T helper cell
TILs: Tumor-infiltrating lymphocytes
TIM-3: T cell immunoglobulin and mucin domain-containing protein 3
TKIs: Tyrosine kinase inhibitors
TMB: Tumor mutation burden
TME: Tumor microenvironment
TNBC: Triple-negative breast cancer
TNF: Tumor necrosis factor
TRAEs: Treatment-related adverse events
Tregs: Regulatory T cells
UDP: Uridine diphosphate
UTP: Uridine triphosphate
VCAM1: Vascular cell adhesion molecule 1
VEGF: Vascular endothelial growth factor
